# Loss of ATM causes R-loop–associated transcriptional dysregulation and attenuates the related response to DNA damage

**DOI:** 10.1016/j.jbc.2026.111161

**Published:** 2026-01-14

**Authors:** Katherine R. Westover, Yingzi Hou, Feng Wang, Yilin Wang, Yangping Li, Rachel Seong, Jie Xu, Zhexing Wen, Bing Yao

**Affiliations:** 1 Department of Human Genetics, School of Medicine, Emory University, Atlanta, Georgia, USA; 2 Department of Psychiatry and Behavioral Sciences, School of Medicine, Emory University, Atlanta, Georgia, USA

**Keywords:** ataxia telangiectasia, ATM, DNA damage response, gene transcription, neurodegenerative disease, neuronal progenitor cells, serine–threonine protein kinase

## Abstract

An early childhood onset neurodegenerative disorder, ataxia telangiectasia (AT), affects one in 40,000 to 100,000 individuals worldwide and is caused by mutations in the ataxia telangiectasia mutated (*ATM*) threonine–serine kinase, which regulates the DNA damage response (DDR). While the cause of AT has been known for years, the exact molecular mechanisms underlying disease progression, particularly at the transcriptomic level, remain poorly understood. Three stranded structures, known as R-loops, have recently emerged as important players in the DDR *via* regulating key gene expression. Here, we utilized neuronal progenitor cells (NPCs) derived from induced pluripotent stem cells reprogrammed from patient-derived somatic cells to identify how loss of ATM impacts R-loop and transcriptional dynamics, both at baseline and in response to acute DNA damage. AT-derived NPCs (AT-NPCs) exhibited elevated spontaneous R-loop levels compared with control-NPCs, as well as a strong positive correlation between R-loop accumulation and gene expression on a subset of dysregulated genes. Upon acute damage, loss of ATM resulted in an attenuated response, characterized by the impaired R-loop and transcriptional response to irradiation. Both control- and AT-NPCs underwent a similar cell cycle arrest, but AT-NPCs displayed an attenuated R-loop and transcriptional response, failing to activate a proper DDR response. Importantly, R-loop formation is required for many key genes to properly respond to DNA damage, supporting a direct and causal role in this process. Overall, our data reveal an underappreciated mechanistic link between ATM, R-loop regulation, and transcription, the disruption of which may contribute to the impaired DDR observed in AT-NPCs.

An early childhood autosomal recessive disorder affecting one in 40,000 to 100,000 individuals worldwide, ataxia telangiectasia (AT), is characterized by progressive loss of motor coordination and movement. A multisystemic disease, AT is caused by mutations in the ataxia telangiectasia mutated (*ATM*) gene, a master regulator of the cell cycle and DNA damage response (DDR) (1). A 350 kDa serine–threonine kinase with a preference for SQ/TQ motifs (2), ATM phosphorylates more than 700 proteins in response to radiation exposure (3). While some individuals show milder phenotypes as a result of leaky splice-site mutations, individuals with highly pathogenic mutations, because of the complete loss of functional ATM, such as a truncation upstream of the kinase domain, develop a severe phenotype (4). AT patients experience a wide range of symptoms, including neurodegeneration, immune defects, increased risk of cancer, radiosensitivity, and chronic lung disease (1). While symptoms may be managed throughout an individual's life, there is currently no effective treatment or cure for AT because of the limited mechanistic understanding of its etiology. Expanding our understanding of the molecular mechanisms underpinning ATM-dependent double-strand break (DSB) repair, particularly at the transcriptomic level, may shed light on identifying promising therapeutic targets for this disease.

It is vital that all cells efficiently and effectively respond to instances of DNA damage through DDR, an essential biological process for cells to maintain genomic stability and continue normal function (5, 6). Improper DDR results in cells being susceptible to apoptosis, genomic instability, a predisposition to cancer, neurodegeneration, and premature aging (7, 8, 9). DDR, therefore, is a crucial network of cellular pathways dedicated to detecting and repairing instances of damage. As a major regulator of DSB repair, ATM is critical for efficient and effective repair (10). Upon experiencing a DSB, the MRE11–RAD50–NBS1 complex recruits ATM to the site of damage and facilitates its activation through monomerization followed by autophosphorylation at serine 367, 1983, and 1981 (11, 12). After activation, ATM then phosphorylates its substrates to initiate the signaling cascade necessary for DNA repair. However, very little is known about how ATM activation transcriptionally regulates key gene expression in a rapid response to DNA damage.

DDR is an intricate dance comprising a multitude of components and requiring precise coordination between functional proteins and genomic structures to control spatial and temporal gene expression (13, 14). One structure that has recently emerged as a critical player in the DDR field is the R-loop. Comprised of an RNA–DNA hybrid with a nontemplate single-stranded DNA, R-loops were initially thought to be transcriptional byproducts with no biological relevance (15). Mounting evidence has demonstrated that R-loops are involved in the regulation of multiple biological processes, such as transcription, class-switch recombination, DNA replication and repair, DNA and histone modifications, among others (16). R-loops are known to have dual roles in transcriptional regulation (17). They can promote transcription when formed over promoters, as DNA methyltransferases preferentially bind to double-stranded DNA over RNA–DNA hybrids, and when formed over CpG islands, R-loops recruit the ten–eleven translocation 1 protein for DNA demethylation (18, 19, 20). When formed downstream of a promoter, or over a gene body, R-loops have been shown to inhibit transcription *in vitro* by causing RNA polymerase II stalling and pausing during transcriptional elongation (21, 22, 23). As the context of the R-loop is vital for its role in transcriptional regulation, dysregulation of these structures may influence the transcriptome and subsequent disease progression.

Recent work has established a direct link between ATM and global R-loop dynamics in response to DSBs. For example, ATM activation upon DNA damage may induce genome-wide R-loop alterations through direct phosphorylation of epigenetic and epitranscriptomic modifiers such as the switch/sucrose non-fermentable remodeler subunit AT-rich interactive domain 1A (ARID1A) and N-6 methyladenosine (m6A) methyltransferase-like 3 (METTL3) (24, 25). These data strongly suggest that ATM-mediated R-loop formation precedes and regulates locus-specific transcription. However, the genome-wide and locus-specific spontaneous R-loop alterations in the context of AT and how they aberrantly respond to DNA damage remain unexplored. A barrier to doing so lies in the inaccessibility of viable human brain tissues for dynamic, functional interrogation. During the past 2 decades, reprogramming of somatic cells into induced pluripotent stem cells (iPSCs) and their downstream cellular models, such as neural progenitor cells (NPCs), has transformed *in vitro* research modeling neurological disorders (26, 27). We have reprogrammed both healthy control and AT patient cells into iPSCs and further differentiated them into NPCs to explore how ATM regulates R-loops and downstream gene expression in AT.

In this study, we evaluate the relationship between ATM loss of function and R-loop regulation, as well as their impact on the transcriptome related to key biological processes such as DDR within control and AT patient–derived NPCs. We show that loss of functional ATM in AT-derived NPCs (AT-NPCs) results in a global increase of R-loops compared with control NPCs (C-NPCs). The upregulated genes with intragenic accumulation of R-loops within AT-NPCs are enriched for neuronal developmental terms, which may indicate aberrant early neurogenesis in AT cells. Through induction of DNA damage by irradiation (IR), we demonstrate that the response in AT-NPCs is impaired compared with the response seen in C-NPCs. Many genes with simultaneous accumulation of R-loops and increased gene expression upon IR in C-NPCs, but not in AT-NPCs, are involved in DDR-related pathways, suggesting a failure to promote those pathways in AT-NPCs. Importantly, R-loop formation is required for many key genes to properly respond to DNA damage, as eliminating global R-loop levels by overexpression of RNase H1 prevents activation of their expression upon IR, supporting a direct and causal role for R-loops in this process. Overall, our results suggest a potential mechanistic role of R-loop regulation in AT pathogenesis and progression because of the loss of functional ATM.

## Results

### Loss of the ATM protein in AT-NPCs results in a global increase in DNA damage and R-loops

**Figure 1:**
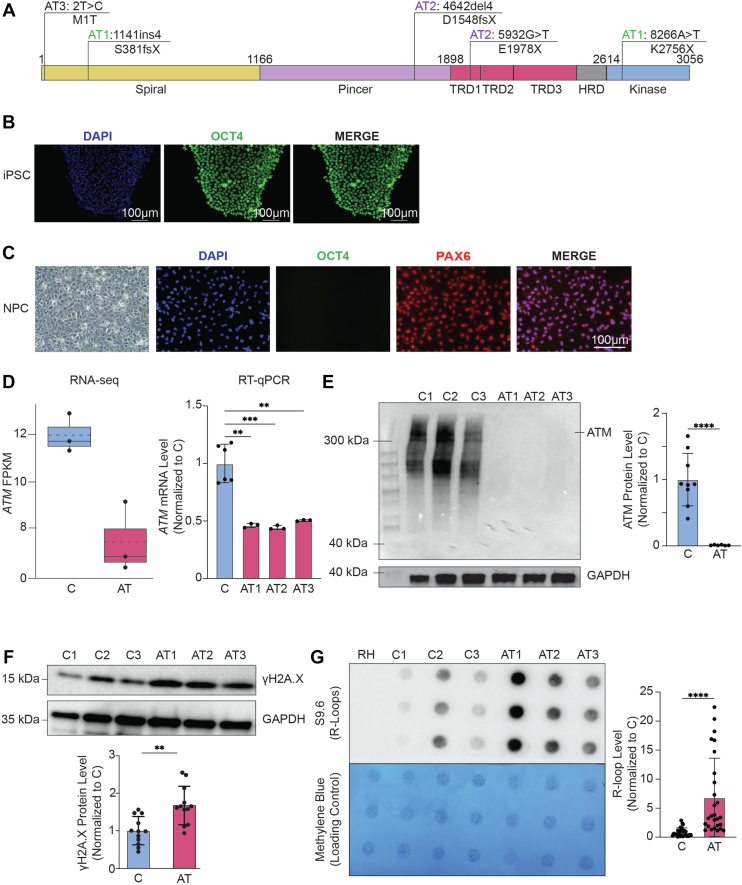
**Loss of the ATM protein in AT patient–derived neuronal progenitor cells (AT-NPCs) results in a global increase in DNA damage and R-loops.***A,* domain diagram of the ATM protein with patient mutations for GM03487 (AT1), GM08436 (AT2), and GM01526 (AT3) obtained from Coriell Biobank is denoted. *B,* representative brightfield and immunofluorescence images showing successful reprogramming of fibroblasts or LCLs into induced pluripotent stem cells (iPSCs). Images were provided by iXCells Biotechnologies. Cells were stained for pluripotency marker OCT4 (*green*). The scale bar represents 100 μm. *C,* representative brightfield and immunofluorescence images showing successful differentiation of iPSCs into NPCs. Cells were stained for pluripotency marker OCT4 (*green*) and NPC marker PAX6 *(red*). The scale bar represents 100 μm. *D,* mRNA expression of *ATM* in both control NPCs (C-NPCs) and AT-NPCs, measured by RNA-Seq (*left*; n = 3) and RT–quantitative PCR (*right*; Student's *t* test). *E,* Western blot analysis of ATM protein levels in C-NPCs and AT-NPCs. Representative Western blot images are shown on the *left*, with quantification of ATM protein levels normalized to GAPDH on the *right*. Quantification data represent three independent experiments, each comparing three C-NPC and three AT-NPC lines (n = 3; Student's *t* test). *F,* Western blot analysis of γH2A.X levels in C-NPCs and AT-NPCs. Quantification of protein levels normalized to GAPDH is shown below (n = 3, Student's *t* test). *G,* dot blot analysis of R-loop levels within C-NPCs and AT-NPCs. Quantification of dots is shown on the *right* (n = 3, Student's *t* test) with methylene blue staining to indicate loading consistency. All data are plotted as mean ± SEM. ∗∗*p* < 0.01; ∗∗∗*p* < 0.001; and ∗∗∗∗*p* < 0.0001. AT, ataxia telangiectasia; ATM, ataxia telangiectasia mutated; FPKM, fragments per kilobase of transcript per million mapped reads; LCL, lymphoblastoid cell line.

To understand how loss of functional ATM may drive R-loop dysregulation, we obtained three AT patient–derived somatic cell lines from the Coriell Biobank. All cell lines tested negative for mycoplasma upon arrival (data not shown). One of the patient cell lines, GM03487 (AT1), was obtained as fibroblasts, whereas the other two, GM08426 (AT2) and GM01526 (AT3), were obtained as lymphoblastoid cell lines (LCLs). We experimentally confirmed that AT1 is a compound heterozygote containing an 8266A>T point mutation resulting in the nonsense mutation K2756X, and a 4-base pair (bp) insertion at position 1141, resulting in the frameshift mutation S381fsX (Figs. 1*A*; S1*A*). AT2 was also a compound heterozygote containing a 5932G>T point mutation resulting in the nonsense mutation E1978X as well as a 4-bp deletion at position 4642, resulting in the frameshift truncation mutation D1548fsX (Figs. 1*A*; S1*A*). AT3 contained a 2T>C mutation, which resulted in the mutation M1T, abolishing initiation (Figs. 1*A*; S1*A*). Mutations in all cell lines were confirmed through Sanger sequencing (Fig. S1*A*). The AT patient–derived cells obtained from Coriell were reprogrammed into iPSCs (see the Experimental procedures section), and successful reprogramming was confirmed by immunostaining for the pluripotency marker OCT4 (Fig. 1*B*). AT- and healthy control–derived iPSCs were then differentiated into neuronal progenitor cells (NPCs, see the Experimental procedures section), with their identities confirmed by the expression of NPC marker PAX6 and no expression of stem cell marker OCT4 (Figs. 1*C*; S1*B*). To confirm ATM deficiency in AT-NPCs, we first assessed *ATM* transcript levels. Both RNA-Seq and RT–quantitative PCR (qPCR) analysis revealed a decrease in *ATM* mRNA within AT-NPCs compared with controls (Fig. 1*D*). Importantly, Western blot analysis showed that ATM protein expression was completely lost in AT-NPCs (Fig. 1*E*), consistent with several other publications using these mutated lines (28, 29, 30, 31).

With the loss of functional ATM, AT-NPCs experienced persistent DNA damage as they were unable to efficiently respond to DSBs (32, 33). We validated this through Western blot and immunofluorescence analysis of the DNA damage marker γH2A.X. We found that AT-NPCs expressed higher levels of γH2A.X protein than C-NPCs (Fig. 1*F*). Quantifying γH2A.X foci in immunofluorescence images confirmed that AT-NPCs had higher numbers of foci per cell compared with C-NPCs (Fig. S1*C*). Given the critical roles of R-loops in the DDR, we next investigated how loss of functional ATM impacts R-loop levels. We performed an R-loop dot blot using the S9.6 antibody and found that R-loop levels were generally elevated in AT-NPCs (Fig. 1*G*). Having established that AT-NPCs demonstrated increased levels of R-loops and DNA damage compared with C-NPCs, we then chose to investigate the specific genomic R-loop changes that result from loss of the ATM protein.

### AT-NPCs demonstrate a higher accumulation of R-loops, which correlate with upregulation of genes involved in neuronal function and DDR

**Figure 2:**
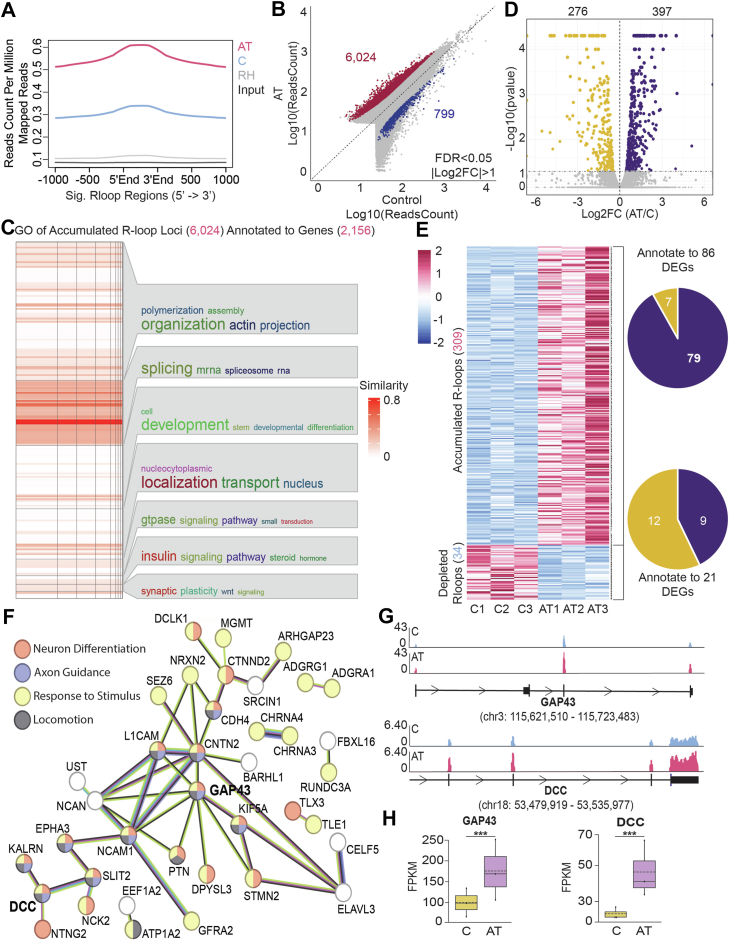
**AT-NPCs demonstrate a higher accumulation of R-loops, which correlates with upregulation of genes involved in neuronal function and DNA damage response.***A,* metagene plot using ngs.plot of R-loop reads over all R-loop regions. *B,* scatterplot of DRIP-Seq results. *Red dots* indicate significantly accumulated R-loops, and *blue dots* indicate significantly depleted R-loops in AT-NPCs (DESeq2, n = 3, FDR < 0.05, |Log_2_FC| > 1). *C,* clustered Gene Ontology (GO) analysis of genes (n = 2156) associated with accumulated R-loop loci (n = 6024) in AT-NPCs. *D,* Volcano plot of RNA-Seq results (Cuffdiff, n = 3, *p* < 0.05). Significantly upregulated genes in AT-NPCs are shown in *purple*, and significantly downregulated genes are shown in *gold*. *E,* heatmap of significantly accumulated (n = 309) and depleted (n = 34) R-loop regions (*left*), with corresponding annotated genes that are significantly differentially expressed (*right*). Pie chart (*top right*) indicates genes that show both accumulated R-loops and significant differential expression, with 79 genes upregulated (*purple*) and seven genes downregulated (*gold*). Pie chart (*bottom right*) indicates genes that show both depleted R-loops and significant differential expression, with nine genes upregulated (*purple*) and 12 genes downregulated (*gold*). *F,* network analysis of the 79 genes with increased levels of R-loops and expression. Node color indicates associated GO terms of the gene (*red* = neuronal differentiation; *blue* = axon guidance; *yellow* = response to stimulus; and *black* = locomotion). *G,* Integrative Genomic Viewer (IGV) browser showing R-loop levels at *GAP43* (*top*) and *DCC* (*bottom*) loci. *H,* RNA-Seq results for *GAP43* (*left)* and *DCC* (*right*) mRNA levels (n = 3; Student's *t* test). All data are plotted as mean ± SEM. ∗∗∗*p* < 0.001. AT-NPC, ataxia telangiectasia–derived neuronal progenitor cell; DCC, Deleted in Colorectal Cancer; DRIP-Seq, DNA–RNA immunoprecipitation sequencing; FC, fold change; GAP43, Growth Associated Protein 43; FDR, false discovery rate; RH, RNase H–treated sample.

To systematically assess genomic R-loop differences between C-NPCs and AT-NPCs, we conducted DNA:RNA immunoprecipitation followed by high-throughput sequencing (DRIP-Seq) to identify differential R-loop regions. We first confirmed that any differences identified were due to cell identity by performing a principal component analysis (Fig. S2*A*). Cell lines clustered separately based on cell identity, confirming that the three AT-NPC lines were more similar to each other than to the three C-NPC lines and *vice versa*. We identified an overall increase of R-loops within AT-NPCs compared with C-NPCs (Fig. 2*A*), which is consistent with what we observed in the dot blot (Fig. 1*F*). Utilizing DESeq2 (34), we identified 6024 accumulated R-loop regions and 799 depleted R-loop regions within AT-NPCs (Fig. 2*B*). We plotted the normalized R-loop reads within these identified regions and confirmed the trend of R-loop changes across these regions (Fig. S2*B*). R-loop peak annotation showed that most R-loop–accumulated regions in AT-NPCs were located within gene bodies (71%), with 16% in intergenic regions and 7% in promoters. A higher percentage of R-loop–depleted regions were localized to intergenic regions (37%), with 52% to gene bodies and 6% to promoters (Fig. S2*C*).

After identifying significantly differential R-loop loci, we next asked how these R-loop changes correlate with functional gene expression. Given that R-loop accumulation is a key feature in AT-NPCs, we focused on the 6024 accumulated R-loop loci and their 2156 intragenic annotated genes. Clustered Gene Ontology (GO) analysis of those 2156 genes revealed significant enrichment for various biological processes, such as RNA splicing and nuclear transport (Fig. 2*C*). The 358 genes associated with R-loop depletion were enriched for developmental GO terms (Fig. S2*D*). To understand the transcriptomic alterations between C-NPCs and AT-NPCs, we performed RNA-Seq on three C-NPCs and AT-NPCs. Using Cuffdiff (35), we found that 397 genes were upregulated in AT-NPCs and 276 were downregulated (Fig. 2*D*). GO analysis of genes upregulated in AT-NPCs revealed an enrichment of neuronal differentiation terms (Fig. S2*E*). Downregulated genes were associated with GO terms such as DDR and regulation of transcription (Fig. S2*F*).

Having established baseline differences in the R-loop and transcriptional landscape between C-NPCs and AT-NPCs, we next sought to identify loci with dysregulation in both R-loops and transcription. The transcriptome was grouped into genes that accumulated R-loops in AT-NPCs, genes that were depleted of R-loops, and genes with no significant differences in R-loop levels between the two cell types. Within each group, we assessed overall changes in gene expression between AT-NPCs and C-NPCs. A half-jitter plot (Fig. S2*G*) showed a general trend of positive correlation between R-loop alteration and gene expression changes, suggesting a potential regulatory link between R-loop dynamics and transcriptional activity. We next identified genes with significant changes in both R-loop levels and gene expression. Three hundred and nine R-loop–accumulated regions were annotated to 86 differentially expressed genes (DEGs; Fig. 2*E*). Among these, 79 (92%) genes exhibited both accumulated R-loops as well as increased gene expression, whereas seven genes showed increased R-loops but decreased expression, confirming a strong positive correlation between R-loop accumulation and gene upregulation. Of the 34 R-loop–depleted regions annotated to 21 DEGs, 12 genes showed decreased expression, whereas nine genes were upregulated. The protein–protein interaction network of the 79 genes with accumulated R-loops and significantly increased gene expression was analyzed using the STRING database (36). Many of these genes are involved in neurogenesis and responding to stimuli, suggesting that AT-NPCs undergo early neurogenesis and DNA damage (Fig. 2*F*). Integrative Genomics Viewer analyses of two representative genes, the neuronal plasticity protein Growth Associated Protein 43 (*GAP43*) (37) and the Netrin-1 (NET1) receptor Deleted in Colorectal Cancer (*DCC*) (38), confirmed higher levels of R-loops at these loci in AT-NPCs compared with C-NPCs (Fig. 2*G*). RNA-Seq also showed that these genes were significantly upregulated in AT-NPCs (Fig. 2*H*). Through this analysis, we have identified a subset of neuronal genes that are differentially expressed in AT-NPCs, potentially caused by changes in the R-loop landscape of these cells.

### Irradiation induces the accumulation of DNA damage and activation of ATM

**Figure 3:**
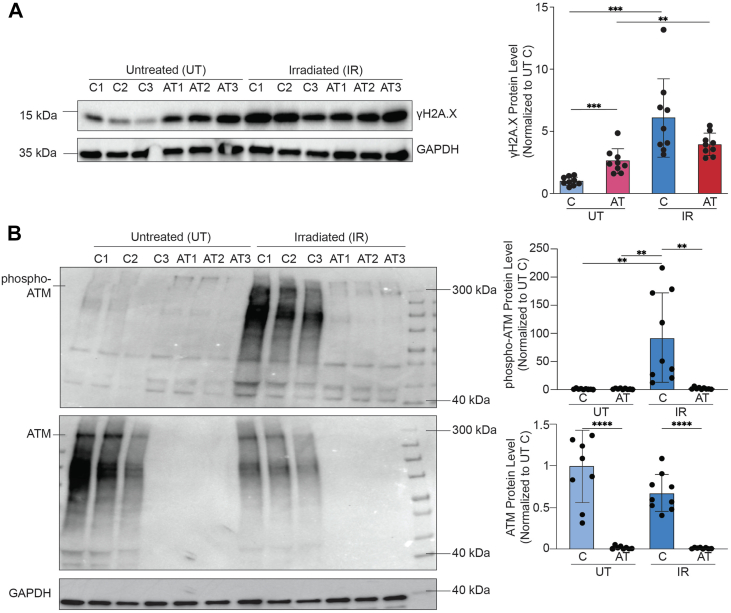
**Irradiation induces the accumulation of DNA damage and activation of ATM.***A,* Western blot of γH2A.X protein levels in untreated (UT) and irradiated (IR) C-NPCs and AT-NPCs. Quantification is shown to the *right* (n = 9; Student's *t* test). *B,* Western blot analysis of S1981 phosphorylated ATM (*top*) and nonphosphorylated ATM (*middle*) in UT and IR C-NPCs and AT-NPCs. Quantifications of protein levels normalized to GAPDH are shown to the *right* (n = 9; Student's *t* test). All data are plotted as mean ± SEM. ∗∗*p* < 0.01; ∗∗∗*p* < 0.001; and ∗∗∗∗*p* < 0.0001. ATM, ataxia telangiectasia mutated; AT-NPC, ataxia telangiectasia–derived neuronal progenitor cell; C-NPC, control NPC.

We next asked how C-NPCs and AT-NPCs respond to acute DNA damage. Cells were exposed to 10 Gy of ionizing radiation to induce DSBs and placed in an incubator for 6 h (39, 40, 41), and untreated (UT) cells were used as controls for comparison. We measured the DDR to IR in C-NPCs and AT-NPCs through γH2A.X Western blots (Fig. 3*A*) and immunofluorescence imaging (Fig. S3*A*). Both assays confirmed that IR cells had higher levels of γH2A.X compared with UT cells. Interestingly, Western blot analysis revealed a less robust accumulation of γH2A.X upon IR in AT-NPCs compared with C-NPCs, suggesting a potential deficiency in DDR signaling in AT-NPCs, which we investigated next.

ATM is activated upon induction of DSBs through autophosphorylation (12). To assess ATM activation, we performed a Western blot for ATM phosphorylated at serine 1981 (pATM) following IR (Fig. 3*B*). pATM was detected exclusively in IR C-NPCs, confirming ATM activation upon DSB induction. Total (nonphosphorylated) ATM was found in both UT and IR C-NPCs, though at slightly lower levels in IR C-NPCs compared with UT, suggesting that DSBs primarily alter the phosphorylation state of ATM rather than its overall protein levels. In contrast, we did not observe any ATM, either phosphorylated or not, in AT-NPCs, confirming that loss-of-function mutations prevent expression of the ATM protein. We also found activation of the ATR (ATM and Rad3-related kinase) pathway, a DDR pathway, which primarily responds to single-strand breaks but has been shown to partially compensate for loss of ATM (42, 43), in both C-NPCs and AT-NPCs upon IR. We found increased phosphorylation of the cell cycle checkpoint protein checkpoint kinase 1 (CHK1) at S345, specifically phosphorylated by ATR in response to genotoxic stress (44), in all cell lines upon IR (Fig. S3*B*). Both C-NPCs and AT-NPCs demonstrated a similar ATR response to IR, suggesting that differences between their R-loop and transcriptional responses to damage are due to the absence of functional ATM in AT-NPCs. These findings indicate that IR triggers a response to DNA damage, which is more robust and properly activated in C-NPCs.

### AT-NPCs demonstrate an impaired R-loop response to irradiation

**Figure 4:**
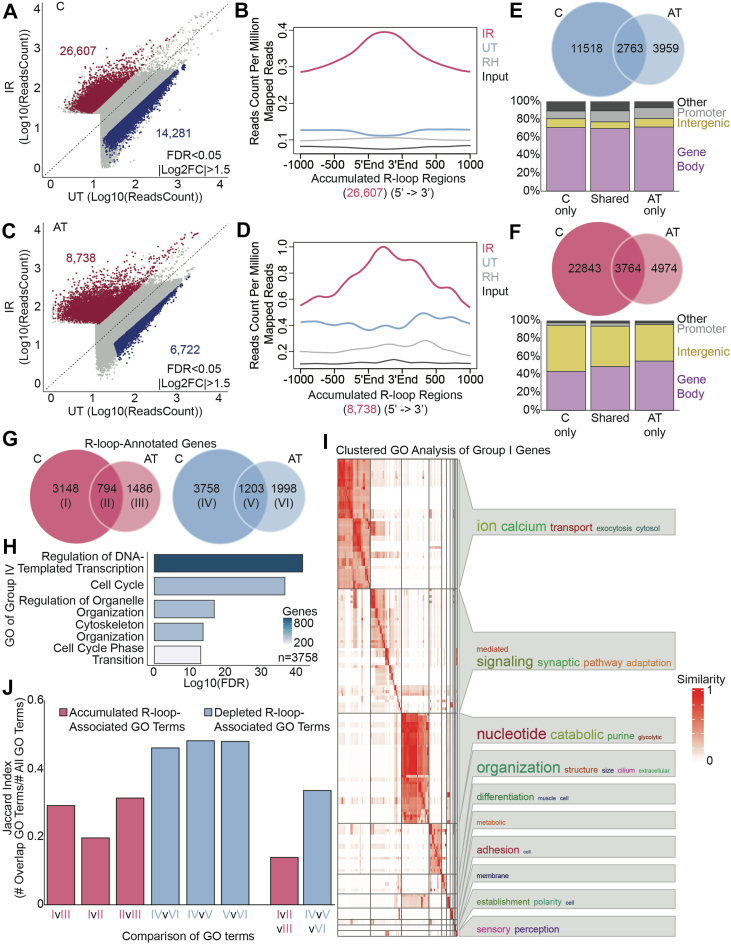
**AT-NPCs demonstrate an impaired R-loop response to irradiation.***A,* scatterplot of DRIP-Seq results for C-NPCs. The *red dots* indicate significantly accumulated R-loops, and the *blue dots* indicate significantly depleted R-loops upon irradiation (DESeq2, n = 3, FDR < 0.05, |Log_2_FC| > 1.5). *B,* metagene plot using ngs.plot of R-loop reads over the 26607 R-loop–accumulated regions in irradiated C-NPCs. *C,* scatterplot of DRIP-Seq results for AT-NPCs. The *red dots* indicate significantly accumulated R-loops, and the *blue dots* indicate significantly depleted R-loops upon irradiation (DESeq2, n = 3, FDR < 0.05, |Log_2_FC| > 1.5). *D,* metagene plot using ngs.plot of R-loop reads over the 8738 R-loop gained regions in irradiated AT-NPCs. *E,* Venn diagram of C-specific (*top left*), AT-specific (*top right*), and shared (*top middle*) R-loop–depleted regions upon irradiation. The stacked bar plot below shows the genomic annotation of depleted R-loops across different genomic regions (including gene body, intergenic region, promoter, and other regions). *F,* Venn diagram of C-specific (*top left*), AT-specific (*top right)*, and shared (*top middle*) R-loop–accumulated regions upon irradiation. The stacked bar plot below shows the genomic annotation of accumulated R-loops across different genomic regions (including gene body, intergenic region, promoter, and other regions). *G,* Venn diagrams of genes with associated accumulation of R-loops (*left*) and depletion of R-loops (*right*). Numerals indicate the number of genes within each group, and numerals within the parentheses indicate group identity. *H,* GO analysis of genes within group IV in (*G*). Bar color indicates the number of genes within each GO term. *I,* clustered GO analysis of genes within group I in (*G*). Font size indicates enrichment level. *J,* Jaccard Index analysis of GO terms between groups in (*G*). The Jaccard Index was calculated as the ratio of the number of overlapping GO terms between two groups to the total number of GO terms within both groups. The index indicates how similar each group's GO terms are to each other. A higher index score indicates more similarities between the groups. Comparisons between groups I, II, and III are in *red*, and comparisons between groups IV, V, and VI are in *blue*. AT-NPC, ataxia telangiectasia–derived neuronal progenitor cell; C-NPC, control NPC; DRIP-SeQ, DNA–RNA immunoprecipitation sequencing; FC, fold change; FDR, false discovery rate; GO, Gene Ontology.

We then performed DRIP-Seq on the IR cells to identify genomic responses to acute damage. Through principal component analysis, we confirmed that C-NPCs and AT-NPCs cluster separately based on treatment type (Fig. S4, *A* and *B*). Differential R-loop analysis of IR *versus* UT revealed that both C-NPCs and AT-NPCs demonstrate more R-loop–accumulated regions than R-loop–depleted regions upon IR. In C-NPCs, we identified 26,607 regions with accumulated R-loop signal and 14,281 regions with depleted R-loop signal (Fig. 4*A*). These trends in R-loop accumulation (Fig. 4*B*) and depletion (Fig. S4*C*) were confirmed through plotting normalized reads to these regions by ngs.plot (45). Importantly, AT-NPCs showed a markedly attenuated response to IR, with 8738 regions of accumulated R-loops and 6722 regions of depleted R-loops (Fig. 4*C*). Trends within these accumulated (Fig. 4*D*) and depleted (Fig. S4*D*) regions were also confirmed by ngs.plot. This pattern in R-loop response is consistent with our previous observations that AT-NPCs demonstrate an impaired γH2A.X response to IR (Fig. 3*A*), suggesting an overall impairment in DDR.

To investigate how C-NPCs and AT-NPCs may respond differently to IR, we first compared regions of R-loop depletion across the two cell types. We identified 11,518 regions, which showed R-loop depletion only in C-NPCs, 3959 regions with depletion only in AT-NPCs, and 2763 regions with depletion in both cell types (Fig. 4*E*). Depleted R-loop regions were primarily annotated to gene bodies, whereas intergenic and promoter regions were less affected. Around 71% of C-only depleted regions were annotated to gene bodies, 10% to intergenic regions, and 9% to promoters. Similarly, of the AT-only depleted regions, around 72% were located in gene bodies, 9% in intergenic regions, and 7% in promoters. In regions of shared R-loop depletion between C-NPCs and AT-NPCs, about 70% were located in gene bodies, 7% in intergenic regions, and 12% in promoters. This distribution is similar to the distribution of all R-loops previously identified in UT NPCs (Fig. S2*C*). When we analyzed the R-loop–accumulated regions, we identified 22,834 C-specific regions, 4974 AT-specific regions, and 3764 shared regions (Fig. 4*F*). Unlike the R-loop–depleted regions, these R-loop–accumulated regions were more frequently located in intergenic regions than expected based on the annotation done in UT NPCs (Fig. S2*C*). Of the C-specific accumulated regions, around 52% were located in intergenic regions, 44% in gene bodies, and 3% in promoters. Around 41% of AT-only regions were located in intergenic regions, 55% in gene bodies, and 2% in promoters. Regions with R-loop accumulation in both C-NPCs and AT-NPCs show a similar distribution, with around 45% located in gene bodies, 49% in intergenic regions, and 4% in promoters. The accumulation of R-loops in intergenic regions upon IR may suggest increased R-loop formation at induced DNA damage sites.

We then annotated all differential R-loops to define their genomic roles and found that 3758 genes showed R-loop depletion upon IR only in C-NPCs (group IV), 1998 only in AT-NPCs (group VI), and 1203 in both cell lines (group V; Fig. 4*G*). When performing GO analysis on these three groups, we found that the genes are enriched in similar terms, mostly relating to the cell cycle (Figs. 4*H*; S4, *E* and *F*). This suggests that IR causes depletion of R-loops within cell cycle–related genes, potentially contributing to cell cycle arrest before DNA repair. Of the genes associated with R-loop accumulation, 3148 showed an accumulation of R-loops in response to IR in C-NPCs only (group I), 1486 genes demonstrated an AT-specific response (group III), and 794 genes had R-loop accumulation in both cell types (group II; Fig. 4*G*). Clustered GO analyses of groups I, II, and III revealed that group I genes had the most GO clusters of the three groups (Fig. 4*I*). Groups II and III genes did not cluster into many groups and had fewer GO terms overall (Fig. S4, *G* and *H*). We then analyzed the similarities of R-loop–associated biological processes affected by IR across different groups using the Jaccard Index, which compared the overlap between groups against the union of the groups, that is, the number of shared GO terms relative to the total number of GO terms in the groups. A higher Jaccard Index indicated greater similarity in enriched GO terms. GO terms for R-loop–depleted gene sets (groups VI, V, and IV; Fig. S4*I*) and for R-loop–accumulated gene sets (groups I, II, and III; Fig. S4*J*) were compared for this analysis. We found that GO terms associated with R-loop–depleted genes had a higher Jaccard Index, indicating greater similarity between these groups, compared with the more divergent GO terms observed in R-loop accumulation groups (Fig. 4*J*). These results suggest that both C-NPCs and AT-NPCs undergo a similar cell cycle arrest response upon IR. However, insufficient R-loop accumulation in AT-NPCs may impair an effective transcriptional response to damage, potentially resulting in disease progression.

### AT-NPCs lack R-loop–associated changes in transcription in DDR-related genes

**Figure 5:**
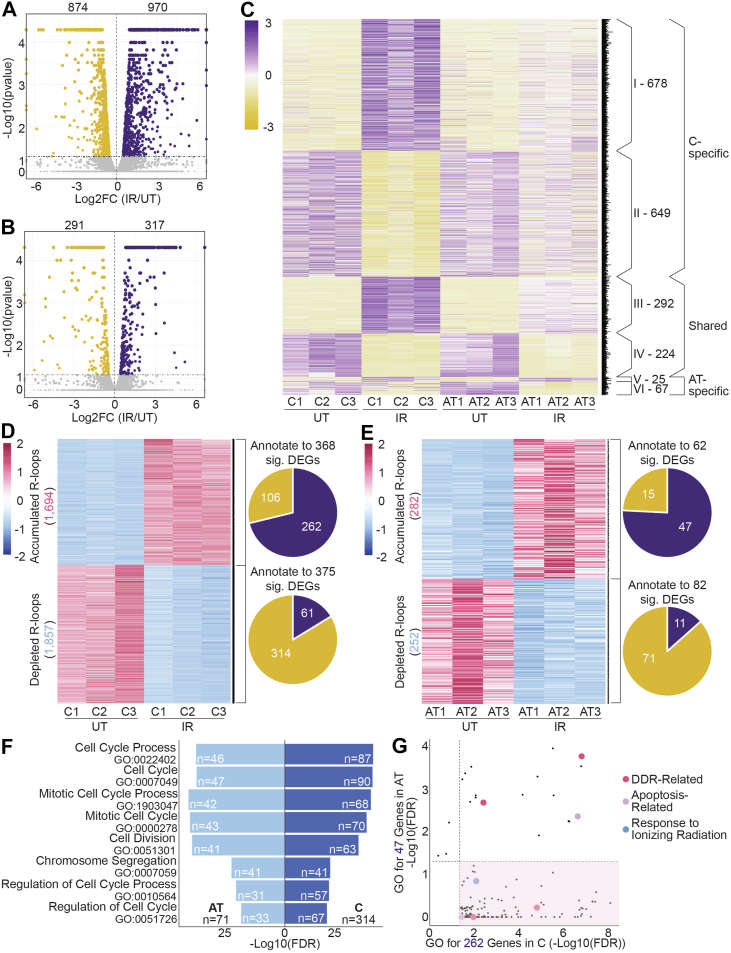
**AT-NPCs lack R-loop–associated changes in transcription in DDR-related genes.***A,* Volcano plot of RNA-Seq results (Cuffdiff, n = 3, *p* < 0.05) for C-NPCs. Significantly upregulated genes upon irradiation are shown in *purple*, and significantly downregulated genes are shown in *gold*. *B,* Volcano plot of RNA-Seq results (Cuffdiff, n = 3, *p* < 0.05) for AT-NPCs. Significantly upregulated genes upon irradiation are shown in *purple*, and significantly downregulated genes are shown in *gold*. *C,* heatmap of significantly differentially expressed genes upon irradiation in C-NPCs and AT-NPCs. Numerals indicate the number of genes in each expression pattern group. Numbers indicate the number of genes within each group. *D,* heatmap of significantly accumulated (n = 1694) and depleted (n = 1857) R-loop regions in C-NPCs upon irradiation (*left*), with corresponding annotated genes that are significantly differentially expressed (*right*). Pie chart (*top right*) indicates genes that show both accumulation of R-loops and significant differential expression, with 262 genes upregulated (*purple*) and 106 genes downregulated (*gold*). Pie chart (*bottom right*) indicates genes that show both depletion of R-loops and significant differential expression, with 61 genes upregulated (*purple*) and 314 genes downregulated (*gold*). *E,* heatmap of significantly accumulated (n = 282) and depleted (n = 252) R-loop regions in AT-NPCs upon irradiation (*left*), with corresponding annotated genes that are significantly differentially expressed (*right*). Pie chart (*top right*) indicates genes that show both accumulation of R-loops and significant differential expression, with 47 genes upregulated (*purple*) and 15 genes downregulated (*gold*). Pie chart (*bottom right*) indicates genes that show both depletion of R-loops and significant differential expression, with 11 genes upregulated (*purple*) and 71 genes downregulated (*gold*). *F,* GO analysis of genes with both significant downregulation and R-loop depletion in C-NPCs (n = 314; *right*) and AT-NPCs (n = 71; *left*) upon irradiation. The number of genes associated with each GO term is marked within the bar. *G,* comparison of significantly enriched GO terms (FDR < 0.05) for genes with both significant upregulation and R-loop accumulation in C-NPCs (n = 262; *x*-axis) and AT-NPCs (n = 47; *y*-axis) upon irradiation. GO terms related to the DDR, apoptosis, and response to ionizing radiation are highlighted in *red*, *purple*, and *blue*, respectively. GO terms significantly enriched only in C-NPCs are highlighted by the *pink box*. AT-NPC, ataxia telangiectasia–derived neuronal progenitor cell; C-NPC, control NPC; DDR, DNA damage response; FDR, false discovery rate; GO, Gene Ontology.

To understand how the dysregulation of the R-loop response to IR may impact transcription and therefore disease progression in AT-NPCs, we performed RNA-Seq in the IR cells. Similar to the observed γH2A.X and R-loop patterns shown earlier, AT-NPCs demonstrated an impaired transcriptional response upon IR. While C-NPCs showed an upregulation of 970 genes and downregulation of 847 genes (Fig. 5*A*), the AT-NPC response was impaired, with only 317 genes upregulated and 291 downregulated (Fig. 5*B*). GO analysis of genes upregulated in C-NPCs upon IR revealed an enrichment of repair-associated GO terms, such as response to stress, cell death, and DDR (Fig. S5*A*). Similar analysis of AT-NPCs' upregulated genes revealed similar terms but with fewer genes involved in each pathway (Fig. S5*C*). Interestingly, GO analyses of genes downregulated in C-NPCs and AT-NPCs both showed enrichment for cell cycle–related terms (Fig. S5, *B* and *D*), although AT-NPCs again had fewer genes associated with each term. This attenuated response parallels what we observe in the R-loop and γH2A.X response (Figs. S5, *A*, *B*, *C*, and *D*; 3*A*). We then compared DEGs across C-NPCs and AT-NPCs and identified six distinct groups of transcriptional responses to IR (Fig. 5*C*). Most DEGs only demonstrated a response to IR in C-NPCs, with 678 (group I) genes upregulated and 649 (group II) genes downregulated. Only 25 genes (group V) were upregulated, and 67 genes (group VI) were downregulated in an AT-specific manner. Two hundred ninety-two genes (group III) were upregulated, and 224 genes (group IV) were downregulated in both C-NPCs and AT-NPCs.

After identifying patterns in the transcriptomic response to IR in C-NPCs and AT-NPCs, we next overlapped our RNA-Seq data with DRIP-Seq data. For both C-NPCs and AT-NPCs, we grouped the transcriptome into genes that accumulated R-loops upon IR, genes that were depleted of R-loops, and genes that showed no significant R-loop response to IR. We then explored the differential expression of the genes in each category. While we saw a general positive correlation between R-loop changes and differential expression, the correlation is weaker in AT-NPCs (Fig. S5*E*) than in C-NPCs (Fig. S5*F*). We also found that fewer genes contain significantly accumulated (1646) and depleted (2118) R-loops in AT-NPCs compared with C-NPCs (3050 and 3103, respectively) (Fig. S5, *E* and *F*). In C-NPCs, we identified 1694 significantly accumulated R-loop regions, which were annotated to 368 genes with significantly differential expression. Of these genes, 262 showed significant accumulation of R-loops and significant upregulation, whereas 106 showed significant downregulation (Fig. 5*D*). Of the 375 genes with significant R-loop depletion, 61 demonstrated significant upregulation and 314 significant downregulation. These results again emphasize a strong positive correlation between R-loop alteration and expression changes. In AT-NPCs, 282 significantly accumulated R-loop regions were annotated to 62 DEGs, with 47 significantly upregulated and 15 significantly downregulated (Fig. 5*E*). We identified 252 significantly depleted R-loop regions, which were annotated to 82 significantly DEGs. Of these genes, 11 were significantly upregulated and 71 significantly downregulated upon IR. Together, these data demonstrate a more subdued overlapped response in AT-NPCs.

We then analyzed genes that demonstrated both R-loop depletion and downregulation and identified multiple shared terms between C-NPCs and AT-NPCs, many of which were cell cycle related (Fig. 5*F*), consistent with our GO analyses of genes downregulated upon IR (Fig. S5, *B* and *D*). While the GO terms were shared between the two cell types, AT-NPCs demonstrated a lower number of genes per term, suggesting an impaired response. To explore what pathways genes with both significant R-loop accumulation and upregulation were enriched for, we performed GO analysis on these genes, as well as on upregulated genes with no associated R-loop changes, in both C-NPCs and AT-NPCs. In C-NPCs, GO terms related to DDR and response to ionizing radiation were significantly enriched (false discovery rate [FDR] < 0.05), specifically in the R-loop–associated gene set but not in the non–R-loop-associated genes (Fig. S5*G*). While some apoptosis-related terms were only enriched in non–R-loop-associated genes, the majority of them were also enriched in both categories (Fig. S5*G*). Overall, this may suggest that in C-NPCs, R-loop accumulation plays a significant role in promoting the transcriptional activation of DDR pathways, which is lost in AT-NPCs. Instead, genes with significant upregulation but no associated R-loop accumulation were more enriched for DDR terms, indicating a loss of R-loop–mediated transcriptional regulation upon IR in AT-NPCs (Fig. S5*H*). When we compared GO terms of R-loop–associated upregulated genes between C-NPCs and AT-NPCs, we found that terms related to DDR, apoptosis, and response to ionizing radiation were significantly enriched in C-NPCs, with only a few DDR- and apoptosis-related terms also enriched in AT-NPCs (Fig. 5*G*). This suggests that the loss of proper R-loop response to IR in AT-NPCs may result in the failure to turn on genes involved in damage response pathways. Overall, these data suggest that loss of functional ATM results in a diminished R-loop–mediated transcriptional response to DNA damage. As a result, R-loop–associated genes, many of which are involved in DDR pathways, are not properly regulated, potentially contributing to disease progression in AT.

### R-loops play broad and causal roles in responding to DNA damage induced from diverse sources, by regulating the expression of a subset of key DDR genes

**Figure 6:**
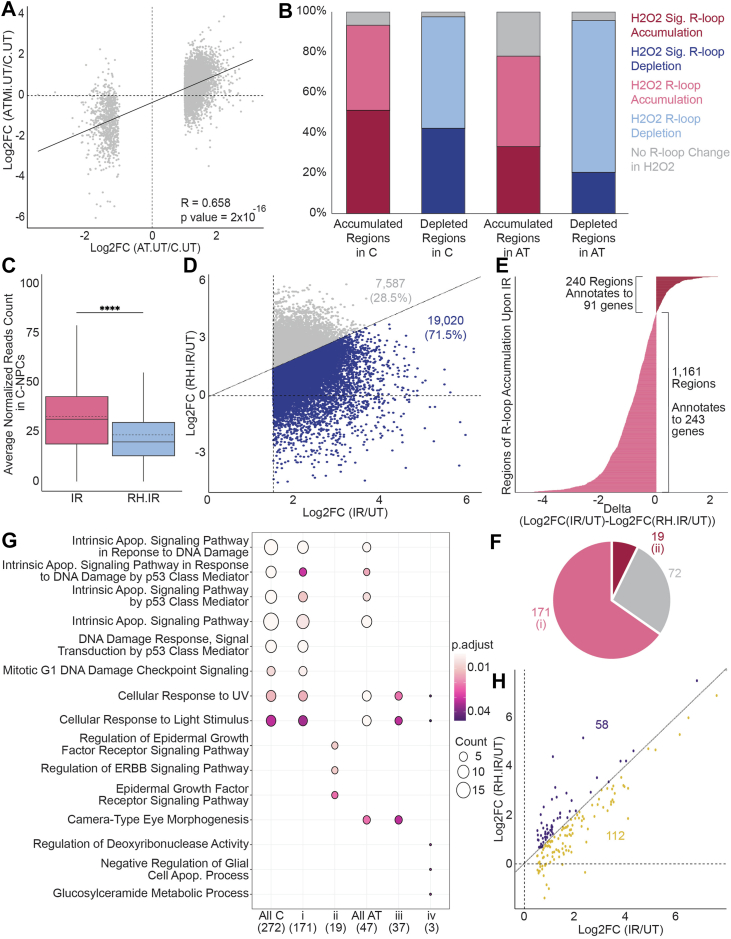
**R-loops play broad and causal roles in responding to DNA damage induced from diverse sources by regulating the expression of a subset of key DDR genes.***A,* scatterplot of DRIP-Seq results of genetic loss (*x*-axis) and chemical inhibition (*y*-axis) of ATM compared against control cells. Correlation between the two comparisons indicated by the *black line* (DESeq2, n = 3, R = 0.658, *p* = 2 × 10^−16^). *B*, stacked bar plot analysis of regions of significant R-loop change (FDR < 0.05 and |Log_2_FC| > 1.5) in irradiation in NPCs overlapped with R-loop response to H_2_O_2_ (DESeq2, n = 3). Color indicates consistency of H_2_O_2_ results with IR results. Darker colors indicate regions of significant R-loop change upon H_2_O_2_ treatment (FDR < 0.05 and |Log_2_FC| > 1.5). Lighter colors indicate regions of nonsignificant R-loop change (Log2FC ≠ 0). *Gray* indicates regions of nonconsistent change between IR and H_2_O_2_. *C,* boxplot of average normalized read counts (DESeq2, n = 3; Student's *t* test) in irradiated C-NPCs (IR; *pink*) and irradiated C-NPCs overexpressing RNase H1 (RH.IR; *blue*). *D,* scatterplot of Log_2_FC of DRIP-Seq results (DESeq2, n = 3) in regions of R-loop accumulation in IR (Log_2_FC [IR/UT]; *x*-axis) and RH.IR cells (Log_2_FC [RH.IR/UT]; *y*-axis). *Blue dots* indicate regions of dampened R-loop accumulation in response to irradiation in RH.IR cells (19020 regions, 71.5%; Log_2_FC [RH.IR/UT] < Log_2_FC [IR/UT]). *Gray dots* indicate regions of increased R-loop accumulation in response to irradiation in RH.IR cells (7587 regions, 28.5%; Log_2_FC [RH.IR/UT] > Log_2_FC [IR/UT]). *E,* bar plot of the difference between Log_2_FC (RH.IR/C.UT) and Log_2_FC (IR/UT) in regions of significant R-loop accumulation upon irradiation in C-NPCs, which annotate to upregulated genes (n = 1401 regions; delta = Log_2_FC [IR/UT] - Log_2_FC [RH.IR/UT], *x*-axis). *Pink bars* indicate regions of dampened R-loop accumulation in RH.IR cells (1161 regions annotating to 243 genes; delta < 0). *Red bars* indicate regions of increased R-loop accumulation in RH.IR cells (240 regions annotating to 91 genes; delta > 0). *F,* pie chart of genes identified in (*E*). *Pink* indicates genes that contain regions of dampened R-loop accumulation upon irradiation in RH.IR cells (n = 171; group i). *Red* indicates genes that contain regions of increased R-loop accumulation upon irradiation in RH.IR cells (n = 19; group ii). *Gray* indicates genes that contain both regions of dampened accumulation and regions of increased accumulation (n = 72). *G,* bubble plot GO analysis of genes within groups i, ii (6*F*), iii, and iv (S6*G*). Color indicates significance, and circle size indicates the number of genes. *H,* scatterplot of Log_2_FC of RNA-Seq results (CuffDiff, n = 3) in genes with dampened R-loop accumulation upon RNase H1 overexpression in IR (Log_2_FC [IR/UT]; *x*-axis) and RH.IR (Log_2_FC [RH.IR/UT]; *y*-axis). *Gold dots* indicate genes that contain regions of dampened R-loop accumulation and dampened gene upregulation upon irradiation in RH.IR cells (n = 112; Log_2_FC [RH.IR/UT] < Log_2_FC [IR/UT]). *Purple dots* indicate genes that contain regions of dampened R-loop accumulation, yet maintain upregulation of gene expression to levels observed in IR cells (n = 58; Log_2_FC [RH.IR/UT] > Log_2_FC [IR/UT]). All data are plotted as mean ± SEM. ∗∗∗∗*p* < 0.0001. ATM, ataxia telangiectasia mutated; C-NPC, control neuronal progenitor cell; DDR, DNA damage response; DRIP-Seq, DNA–RNA immunoprecipitation sequencing; FC, fold change; FDR, false discovery rate; GO, Gene Ontology; H_2_O_2_, hydrogen peroxide; IR, irradiation.

To validate that the dysregulation of R-loops observed in AT-NPCs is due to loss of functional protein rather than potential confounding mutations introduced during the reprogramming and differentiation process, we treated each C-NPC line with an ATM-specific inhibitor, AZD1390 (ATMi). Treating C-NPCs with 100 nM of ATMi for 6 h prevented phosphorylation of ATM upon IR without impacting total ATM levels (Fig. S6*A*). This loss of ATM activity mirrors what we observed happening in AT-NPCs upon IR (Fig. 3*B*). After validating ATMi activity, we then investigated if the R-loop landscape in control cells treated with 100 nM of ATMi for 12 h (ATMi-NPCs) resembled that of AT-NPCs. We had previously defined regions of significant R-loop accumulation and depletion within AT-NPCs compared with C-NPCs (Fig. 2*B*). The R-loop response within these predefined regions was compared between our ATMi-NPCs and AT-NPCs, using C-NPCs as a reference. We found a significant (*p* = 2.2 × 10^−16^) positive correlation between cells with a genetic loss (AT-NPCs) and cells with a chemical inhibition (ATMi-NPCs) of ATM, with a correlation coefficient of 0.658 (Fig. 6*A*). This correlation indicates that R-loops within our AT- and ATMi-NPCs act in a similar manner and validates that the R-loop response observed in AT-NPCs is due to loss of functional ATM.

We also investigated if the R-loop response observed is specific to IR-induced damage or if it is a general response to DSBs. To address this, we treated all C-NPCs and AT-NPCs with 1 mM hydrogen peroxide (H_2_O_2_), a chemical that induces DSBs through the production and response to hydroxyl radicals, a reactive oxygen species (46), for 6 h. We first identified regions of significant R-loop accumulation and depletion upon H_2_O_2_ treatment in C-NPCs and AT-NPCs and compared these with regions of significant R-loop changes upon IR (Fig. S6*B*). Overall, we found that upon induction of DNA damage in C-NPCs, 31% of IR regions and 41% of H_2_O_2_ regions overlapped. When analyzing regions of R-loop depletion, 42% of IR regions and 31% of H_2_O_2_ regions were shared (Fig. S6*B*). This suggests that under normal conditions, a subset of R-loops is part of a general response to DNA damage, whereas others respond in an induction-specific manner. This general damage response in C-NPCs is impaired in AT-NPCs, with only 17% of IR accumulation regions and 14% of H_2_O_2_ accumulation regions shared between the two treatments. When investigating regions of R-loop depletion, 20% of IR regions and 19% of H_2_O_2_ regions overlapped (Fig. S6*B*). The loss of ATM may cause AT-NPCs to use different compensatory mechanisms to respond to the two methods of DNA damage induction. We also analyzed the regions of significant R-loop accumulation in C-NPCs upon IR and found that around 51.2% of these regions also demonstrated a significant accumulation of R-loops (Log_2_ fold change > 1.5 and FDR < 0.05) upon H_2_O_2_ treatment around 42.3% of regions showed nonsignificant accumulation of R-loops (Log_2_ fold change > 0), and 6.5% showed no R-loop accumulation (Fig. 6*B*). Of the R-loop–depleted regions in C-NPCs, around 42.3% also demonstrated significant depletion, 55.4% showed nonsignificant levels of depletion, and 2.3% did not show R-loop depletion. This overlap was dampened in AT-NPCs, as only 33.2% of regions of R-loop accumulation showed significant accumulation upon H_2_O_2_ treatment, 45% showed nonsignificant R-loop accumulation, and 21.8% showed no accumulation (Fig. 6*B*). Of the R-loop–depleted regions upon IR, 20.5% of these regions showed significant depletion in H_2_O_2_-treated AT-NPCs, 75.4% showed nonsignificant levels of depletion, and 4.1% showed no depletion. Overall, these data suggest that when the ATM is functional, R-loops demonstrate both an induction-specific and a general damage response. This general damage response is lost when the ATM is nonfunctional, leading to compensatory mechanisms operating through different pathways depending on the method of DSB induction.

We next worked to determine the hierarchical order and mechanistic causality between R-loops and transcription in response to DNA damage. FLAG-tagged RNase H1 was overexpressed in NPCs upon lentiviral infection. Forty-two hours postinfection, cells were irradiated with 10 Gy. Utilizing a multiplicity of infection of 5, we were able to express the FLAG-RNase H1 construct within NPCs (Fig. S6*C*). Upon RNase H1 overexpression, we observed lower R-loop read counts in both IR C-NPCs (Fig. 6*C*) and IR AT-NPCs (Fig. S6*D*), confirming that RNase H1 overexpression leads to a global decrease in R-loops. We then compared both IR NPCs (IR) and NPCs overexpressing RNase H1 (RH.IR) to UT NPCs. We found that in C-NPCs, 71.5% of regions, which demonstrated a significant accumulation upon IR, had a dampened response when the IR cells overexpressed RNase H1, and only 28.5% of those regions showed a comparable or stronger response (Fig. 6*D*). This vulnerability to RNase H1 is even more prominent in AT-NPCs, where 84.3% of regions showed a dampened R-loop response in RH.IR cells (Fig. S6*E*).

We further investigated the regions of R-loop accumulation upon IR in C-NPCs, which were annotated to upregulated genes (1401 regions, Fig. 5*D*) and found that the majority of these regions (1161 regions, 82.9%) also demonstrated a dampened response, with higher accumulation levels found in IR cells compared with RH.IR cells and 240 regions (13.1%) demonstrated a stronger R-loop response (Fig. 6*E*). These 1161 regions of dampened R-loop accumulation were annotated to 243 genes, whereas the 240 regions, which showed increased R-loop response, were annotated to 91 genes. Of these genes, 72 contained both dampened and increased R-loop regions, 171 (group i) contained only regions of dampened accumulation, and 19 (group ii) contained only regions of increased accumulation (Fig. 6*F*). This trend of loss of R-loop response to IR upon RNase H1 overexpression is further exacerbated in AT-NPCs. Of the R-loop regions that showed significant accumulation of R-loops and increased gene expression upon IR in AT-NPCs (242 regions, Fig. 5*E*), 208 of these regions (86%) showed a dampened R-loop response and 34 regions (14%) contained increased accumulation of R-loops (Fig. S6*F*). The 208 regions of dampened accumulation were annotated to 44 genes, and the 34 regions of increased accumulation were annotated to 10 genes (Fig. S6*F*). Seven of these genes contained regions of both dampened and increased R-loop response, 37 (group iii) contained only regions of dampened response, and 3 (group iv) contained only regions of increased response (Fig. S6*G*). When performing a GO analysis on genes which demonstrated a differential R-loop response to IR upon RNase H1 overexpression (groups i–iv), we found that in C-NPCs, genes with an impaired R-loop response (group i) were enriched in DDR-related pathways, whereas the genes not sensitive to RNase H1 (group ii) were enriched in growth signaling pathways (Fig. 6*G*). In AT-NPCs, this correlation between R-loops and DDR is lost, as genes with an impaired R-loop response (group iii) were enriched in environmental response pathways and not DDR pathways (Fig. 6*G*). When we investigated the response to IR within genes with a dampened R-loop response, we found that two-thirds of these genes (112/171) in C-NPCs also show an impaired gene upregulation, suggesting that a subset of DDR genes are regulated by R-loops upon induction of DNA damage (Fig. 6*H*). When we investigated the RNase H1–sensitive genes in AT-NPCs, we lost the correlation between R-loops and transcription, with only 37.8% of genes (14/37) demonstrating a reduction in both R-loops and transcription (Fig. S6*H*).

Overall, these data strongly suggest that a subset of the key genes that transcriptionally respond to IR is regulated by accumulation of R-loops within these genes, and disruption of proper R-loop formation by RNase H1 overexpression has a direct and causal impact on the failure to activate key transcriptional programs required for effective damage control in normal cells, which is further exacerbated upon loss of functional ATM.

## Discussion

In this study, we have begun elucidating the critical relationship between ATM, R-loops, and transcription in the DDR. We have utilized a unique model system of NPCs derived from iPSCs reprogrammed from AT-patient somatic cells to systematically characterize the R-loop landscape in AT and gained a more comprehensive understanding of how loss of ATM impacts R-loop regulation. Overall, we observed increased levels of γH2A.X within AT-NPCs through Western blot and immunofluorescence analysis. These increased levels of γH2A.X were matched by increased R-loop accumulation. A subset of neuronal genes exhibits both a significant accumulation of R-loops as well as a significant upregulation. The neuronal plasticity protein, GAP43, while mainly known for its role in nerve growth, has recently been found to be elevated within the cerebrospinal fluid of Alzheimer's disease patients, where it is associated with a faster spread of amyloid-beta–associated tau accumulation (37, 47). While GAP43 has yet to be studied in AT, its emerging role in neurodegeneration may be interesting to explore in the context of this disease. DCC, when bound by NET1, acts as a guidance cue to promote axon genesis (38). While DCC generally works in concordance with NET1 to promote axon genesis and cell survival, in the absence of sufficient NET1 for binding, DCC has been shown to promote apoptosis (48). The upregulation of DCC without matching upregulation of NET1 (data not shown) in AT-NPCs may therefore contribute to apoptosis within these cells. Our analysis has identified potential contributors to neurodegeneration caused by R-loop dysregulation.

**Figure 7:**
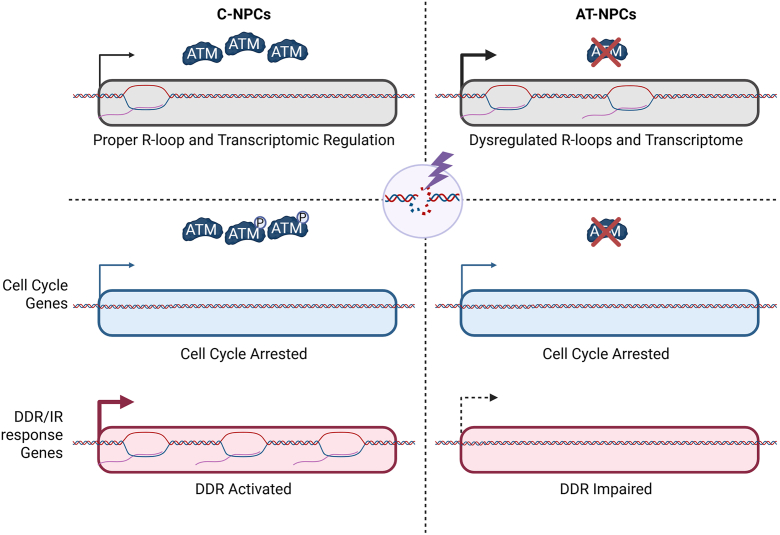
**Loss of ATM causes R-loop–associated transcriptional dysregulation and attenuates the related response to DNA damage.** ATM, ataxia telangiectasia mutated.

Upon induction of acute DNA damage through IR, we observed increased DDR levels, shown through increased levels of γH2A.X, as well as activation of ATM in C-NPCs. In this study, we identified a much more robust response to IR within C-NPCs in both transcription and R-loop levels. Importantly, we show that a subset of these response genes is regulated by the accumulation of R-loops, as loss of accumulation because of RNase H1 overexpression results in an impaired transcriptional response, which is further exacerbated in AT-NPCs. These data provide solid evidence supporting the causal roles of R-loops in dictating key gene expression in response to DNA damage, rather than reflecting a mere consequence of transcription changes. Our data suggest that both C-NPCs and AT-NPCs undergo a similar cell cycle arrest, but that AT-NPCs demonstrate an insufficient R-loop response, which may impact the transcriptional activation of DDR pathways and thus contribute to disease progression (Fig. 7). Our data provide the first glimpse into the dynamic relationship between R-loop regulation, ATM, and transcription and how loss of functional ATM results in dampened DDR in AT-NPCs.

AT is a devastating disease with no current cure caused by mutations within the *ATM* gene. Functional ATM is critical for proper DNA damage repair. Biallelic loss of ATM has been characterized by cerebellar ataxia, hypersensitivity to radiation, immune defects, and increased risk for cancer among other symptoms (49, 50, 51). While a subset of ATM mutations, such as leaky splice site mutations, allows for the protein to retain some function and therefore results in a milder phenotype, nonsense mutations or mutations that result in a truncation of the protein before its kinase domain cause a severe phenotype (4, 49). Recent work has begun utilizing prime editing to identify the functions of *ATM* single nucleotide variants (52). It confirmed that nonfunctional variants show reduced levels of ATM phosphorylation after DNA damage and that nonfunctional single nucleotide variants result in significantly higher cancer incidence (52), suggesting that these types of mutations cause a more severe clinical phenotype in AT patients. ATM loss of function through the use of ATM inhibitors has also been shown to increase R-loop levels within neuron-like cells, which overlap with sites of DNA damage, such as sites of poly (ADP-ribose) signals, potentially caused by increased reactive oxygen species within the cells. Interestingly, ATM inhibition in neuron-like cells does not cause a significant transcriptional change, although that may be due to the relatively short duration of ATM inhibition treatment, reflecting an acute loss of function, or a lack of DNA damage induction. AT patient–derived brain samples, however, do demonstrate dramatic transcriptional changes compared with control brain tissue, which may be due to a chronic loss of functional ATM (53). In our study, we investigated how nonfunctional mutations impact ATM levels within patient-derived NPCs and how the loss of ATM further dysregulates R-loops and transcription.

Failure of proper DSB repair results in the accumulation of DNA damage, genomic instability, and even cell death (54). Upon DSBs, the MRE11–RAD50–NBS1 complex recruits and facilitates the activation of ATM, resulting in a signaling cascade responsible for DNA repair (10, 55). While its regulatory role in DDR is well established, the specific mechanisms through which ATM accomplishes this have yet to be fully explored. Recent studies have begun exploring a potential relationship between R-loop regulation and ATM in DDR (24, 25). While originally thought to simply be byproducts of transcription with no biological relevance (15), R-loops have emerged as important regulators of multiple biological processes, including transcription and DDR. Although R-loops can act as a source of DNA damage, they can also promote homologous recombination (HR) over nonhomologous end joining when formed as a result of DSBs in transcriptionally active genes (56, 57, 58, 59, 60, 61). DSBs at these sites often result in the stalling of RNA polymerase II, which increases the chances of R-loop formation (62). These R-loops can then serve as a recruitment site for DDR proteins such as RAD52 through the binding of Cockayne syndrome protein B, therefore promoting HR (20, 56, 63, 64). Cockayne syndrome protein B directly binds to RNA–DNA hybrids, recruiting RAD52 and therefore mediating the DNA-to-DNA interaction needed for HR complementary strand annealing (65). Recruitment of RAD52 also allows for localization of RAD51 at R-loops (63). Proper regulation of R-loops is therefore crucial for efficient repair. In our AT-NPCs, we see a loss of such regulation and an attenuated transcriptional response to acute damage.

ATM substrates, such as METTL3 and ARID1A, have been demonstrated to regulate R-loop stability in response to DSBs. METTL3 has been shown to be phosphorylated by ATM at serine 43 in response to DSBs (24), and through placing the methyl mark m6A on the RNA strand of R-loops, it regulates their stability (66, 67, 68). The RNase H1 endonuclease, which cleaves the RNA strand of R-loops from DNA, thereby resolving the structure, has been shown to preferentially bind to m6A-modified R-loops for R-loop resolution (25, 69). ARID1A, another substrate of ATM, helps facilitate the recruitment of METTL3 to R-loops for m6A deposition (25). Loss of ARID1A has been shown to cause increased accumulation of R-loops (70). Recent work suggests that upon DNA damage, ARID1A is phosphorylated by ATM, promoting its recruitment to chromatin (25). Other ATM targets, such as breast cancer susceptibility gene 1, have been implicated in R-loop regulation (71). ATM may therefore have a similar regulatory role as its substrates on R-loops. Our study has opened an intriguing avenue of investigation into how ATM regulates R-loops and transcription, and subsequent DDR, in AT patient–derived cells. The failure of m6A deposition onto R-loops because of the loss of ATM may cause an impaired R-loop resolution, resulting in the increased levels of R-loops observed within AT-NPCs. We next plan to elucidate the molecular mechanisms underlying this relationship and how m6A, ARID1A, and METTL3 interact to regulate R-loop–associated DDR.

It has become increasingly clear that dysregulation of R-loops can cause immense damage within cells. These structures have become implicated in multiple diseases, ranging from roles in cancer to contributing to neurodegeneration (72, 73). R-loop regulation has emerged as a potential mechanism of neurodegeneration within multiple diseases, including ataxias. In some cases, mutations within RNA–DNA helicases, such as senataxin and RNase H, which resolve R-loops, lead to aberrant accumulation associated with increased levels of DNA damage (74, 75, 76). In other ataxias, trinucleotide repeat expansions promote the accumulation of R-loops over the repeats, which impacts transcriptional regulation of genes within such regions (77, 78). While the role of R-loops in AT has not been fully investigated, this study begins elucidating a potential molecular mechanism underlying AT disease progression. Our results identify a causal relationship between loss of ATM and dysregulation of R-loops and transcription within patient-derived NPCs. By elucidating the role of R-loops and their dysregulation in AT pathology, we may discover a potential therapeutic target for patients with this disease.

### Limitations

While we identified that the majority of R-loops accumulated upon IR are in intergenic regions, we did not investigate the link between these intergenic R-loops and induced DNA damage sites, as the present study focused on establishing the relationship between R-loops and the transcriptome in the presence or absence of functional ATM. Based on previous studies, we speculate that these intergenic R-loops may form at sites of induced damage (56, 57, 79). Future studies will further explore this link and how the presence of R-loops at these sites impacts DDR.

## Experimental procedures

### Cell lines and ATM inhibitor treatments

Three AT patient–derived cell lines, one fibroblast line (Coriell Institute, #GM03487) and two LCLs (Coriell Institute, #GM08436 and #GM01526), were purchased from the Coriell Institute. The fibroblast line was reprogrammed into iPSCs by the Emory Stem Cell Core. The two LCLs were sent to iXCells Biotechnologies for reprogramming. Three healthy iPSC lines were obtained from the laboratory of Dr Zhexing Wen at Emory University. iPSCs were cultured on Matrigel-coated plates (Corning, #354277) in mTeSR1 (STEMCELL Technologies, #85850) at 37 °C and 5% carbon dioxide (CO_2_). iPSCs were then differentiated into NPCs following STEMCELL Technologies Monolayer Culture protocol for STEMdiff SMADi Neural Induction Kit (STEMCELL Technologies, #08581). NPCs were cultured in STEMdiff Neural Progenitor Medium (STEMCELL Technologies, #05833) at 37°C and 5% CO_2_. For IR experiments, cells were irradiated with 10 Gy and placed in an incubator for 6 h at 37 °C and 5% CO_2_. For the H_2_O_2_ experiments, all cell lines were treated with a 1 mM solution of H_2_O_2_ (MilliporeSigma, #H1009) for 6 h in an incubator at 37 °C and 5% CO_2_. To test ATM inhibition, C-NPCs were treated with 100 nM of the ATM inhibitor AZD1390 (Sellcheck Chemicals, #S8680) for 12 h. An RNase H1 overexpression lentivirus was constructed and infected into all cell lines. After infection, cells were incubated at 37 °C and 5% CO_2_ for 42 h and then irradiated with 10 Gy and placed back in the incubator for 6 h.

### RNA extraction

Total RNA was isolated using the TRIzol reagent (Invitrogen, #15596018). Approximately one million cells were lysed by repetitive pipetting in 1 ml of TRIzol. Phase separation was performed by adding 0.2 ml of chloroform (Sigma–Aldrich, #C2432), followed by centrifugation at 20,000g for 15 min at 4 °C. The top aqueous layer (∼500 μl) was transferred to a new tube, and the RNA was precipitated overnight at −80 °C with an equal volume (500 μl) of 100% isopropanol (Fisher Scientific, #AC610080040), 1/10 volume (50 μl) of 3 M sodium acetate (Teknova, #S0297), and 4 μl of 5 mg/ml glycogen (Thermo Fisher, #AM9510). Samples were then centrifuged at 15,000*g* for 20 min at 4 °C, washed twice with 1 ml of 75% ethanol, and dissolved in 50 μl of nuclease-free water.

### Genomic DNA extraction

Genomic DNA (gDNA) extraction was performed as previously described (76, 80). Cells were washed once with PBS (Thermo Fisher, #AM9624) and then pelleted and suspended in 1.6 ml of Tris–EDTA buffer (TE buffer, 10 mM Tris–HCl [pH 8], and 1 mM EDTA). Fifty microliters of 20% SDS (w/v) (Santa Cruz, #sc-24950) and 5 μl of Proteinase K (20 mg/ml; Invitrogen, #EO0492) were added. Samples were incubated at 37 °C overnight. Samples were transferred to 15 ml high-density Maxtract phase lock gel tubes (Qiagen, #129065), and one volume (1.6 ml) of phenol:chloroform:isoamyl alcohol (25:24:1; Sigma–Aldrich, #P2069) was added to each sample. Samples were centrifuged at 1500*g* for 5 min, and the supernatant was transferred to a new 15 ml tube. Nucleic acids were precipitated by adding 1/10 volume of 3 M sodium acetate (pH 5.2; 160 μl) and 2.5 volumes of 100% ethanol (4 ml). Tubes were gently inverted until the DNA fully precipitated and became visible. Precipitate was then transferred to a 1.5 ml tube, washed twice with 80% ethanol, air-dried, and dissolved in 100 μl of TE buffer. DNA was then fragmented with restriction enzymes (BsrGI, EcoRI, HindIII, SspI, and XbaI, 30 units each) (NEB, #3575S, #R3101S, #R3104S, #R3132L, and #R0145S) at 37 °C overnight. The digested nucleic acids were then recovered by phenol–chloroform extraction.

### Dot blots

gDNA was extracted as described in the “gDNA extraction” section. DNA was diluted to a concentration of 25 ng/μl. Five hundred nanograms of gDNA were applied to a membrane (GE Healthcare, #RPN303B) using the Bio-Dot Apparatus (Bio-Rad, #1706545), followed by washing twice with 6x SSC (Quality Biological, #351-003-131). Membranes were left in a vacuum for 15 min to dry and then crosslinked by incubation at 85 °C for 30 min. Membranes were blocked in blocking buffer (5% nonfat dry milk in Tris-buffered saline containing 0.05% Tween-20 [TBST]) for 30 min at room temperature and then incubated in blocking buffer with primary antibodies at 4 °C on a rotating shaker overnight. Membranes were then washed three times with TBST and incubated with horseradish peroxidase–conjugated anti-mouse IgG secondary antibody (CST, #7076P2, 1:5000 dilution) for 60 min at room temperature. R-loop signals were detected by chemiluminescence (GE, #89168-782) and quantified using Image Lab software (Bio-Rad). The primary antibody used was S9.6 anti-DNA–RNA hybrid antibody (Millipore, #MABE1095; 1:5000 dilution).

### Reverse transcription and real-time PCR

Complementary DNA was synthesized from 500 ng of total RNA using SuperScript III Reverse Transcriptase (Invitrogen, #18080051), following the manufacturer's protocol. Quantitative real-time PCR was performed using the SYBR Green–based PCR assay (Quantabio, #95074). Transcript levels were quantified using the 2^−ΔCT^ method and normalized to the housekeeping gene *β-actin*. The primer sets for ATM and β-actin are as follows: ATM_qPCR_FWD, TTGATCTTGTGCCTTGGCTAC; ATM_qPCR_REV, TATGGTGTACGTTCCCCATGT; β-actin_qPCR_FWD, GGCACCCAGCACAATGAAGATCAA; β-actin_qPCR_REV, ACTCGTCATACTCCTGCTTGCTGA.

### Protein isolation and Western blot analysis

NPCs were cultured on 6-well plates, washed with PBS, and lysed in radioimmunoprecipitation assay lysis buffer (Thermo Fisher, #89901) containing 1X cOmplete Protease Inhibitor Cocktail (Sigma–Aldrich, #11836170001). Lysates were incubated on ice for 20 min with occasional vortexing, followed by centrifugation at 15,000 rpm for 10 min at 4 °C to remove the insoluble fraction. 4X Laemmli sample buffer (Bio-Rad, #34229167) containing 10% β-mercaptoethanol (Fisher Scientific, #21985-023) was added to each protein sample and heated at 100 °C for 10 min. Proteins were separated on either 10% or 4% to 15% precast polyacrylamide gels (Bio-Rad, #4561033 and #4561086) and transferred to polyvinylidene difluoride membranes using precut blotting transfer packs (Bio-Rad, #1704156) following the manufacturer's instructions. Membranes were blocked in blocking buffer (5% nonfat dry milk in TBST) for 1 h at room temperature and then incubated in blocking buffer with primary antibodies at 4 °C on a tilting shaker overnight. Membranes were then washed three times with TBST and incubated with horseradish peroxidase–conjugated anti-rabbit or anti-mouse IgG secondary antibodies (CST, #7074S and #7076P2, 1:5000 dilution). Protein signals were detected by chemiluminescence (GE, #89168-782) and quantified using Image Lab software (Bio-Rad). The primary antibodies used were anti-gamma H2A.X antibody (Abcam, #ab81299; 1:1000 dilution), anti-ATM antibody (Abcam, #ab32420; 1:1000 dilution), anti–phospho-ATM antibody (Abcam, #ab81292; 1:50,000 dilution), anti–phospho-checkpoint kinase 1 antibody (Cell Signaling Technology, #2348T; 1:1000 dilution), anti-FLAG antibody (Sigma–Aldrich, #F1804-5MG; 1:3000 dilution), anti–alpha tubulin antibody (Sigma–Aldrich, #T5168-100UL; 1:1000 dilution), and anti-GAPDH antibody (Thermo Fisher, #AM4300; 1:3000 dilution).

### Immunofluorescence microscopy

Cells were cultured on coverslips coated with Matrigel placed in 6-well plates (Thermo Fisher, #140675), rinsed with PBS three times, and fixed in 4% paraformaldehyde (Thermo Fisher, #047392.9M) for 20 min. Following fixation, cells were washed three times with PBS, permeabilized with 0.5% Triton X-100 (Sigma–Aldrich, #T8787) for 15 min, and washed three times again with PBS. Cells were then blocked with 5% normal goat serum (Cole-Parmer, #S-1000) in PBS for 30 min and incubated with primary antibody (1:500 dilution in 5% normal goat serum) overnight at 4 °C. After three PBS washes, cells were incubated with fluorescent secondary antibodies (Invitrogen, #A10042, #A11029) for 2 h at room temperature, followed by nuclear staining with 4′,6-diamidino-2-phenylindole (Thermo Fisher, #62248) for 10 min. After three PBS washes, coverslips were mounted onto glass slides using mounting medium (Thermo Fisher, #00-4958-02). Images were acquired using an all-in-one Fluorescence Microscope (Keyence and LEICA DMi8), and fluorescence intensity was quantified using ImageJ software (developed by the NIH; https://imagej.net/ij/). The following primary antibody were used: anti-gamma H2A.X antibody (Abcam, #ab81299; 1:500 dilution), anti-PAX6 antibody (STEMCELL Technologies, #60094; 1:500 dilution, Fig. 1*C*) (Thermo Fisher, #MA1-109; 1:500 dilution, Fig. S1*B*), and anti-OCT4 antibody (STEMCELL Technologies, #60093; 1:1000 dilution).

### DNA–RNA immunoprecipitation

DRIP was performed as previously described (76, 80). Briefly, gDNA was extracted as described in the “gDNA extraction” section and enzymatically fragmented. Five micrograms of fragmented nucleic acids were treated with 20 U of RNase H (NEB, #M0197L) for 6 h at 37 °C as a negative control. For each DRIP reaction, 5 μg of digested DNA (with or without RNase H treatment) was incubated with 8 μl (∼8 μg) of anti-DNA–RNA S9.6 antibody (Millipore, #MABE1095) overnight at 4 °C in 1X DRIP binding buffer (10 mM NaPO_4_ [pH 7.0], 140 mM NaCl, and 0.05% v/v Triton X-100). DNA:RNA hybrids were captured using Protein G beads (40 μl; Invitrogen, #10009D) by rotating at 4°C for 2 h. Beads were then washed twice with 1X DRIP binding buffer, and DNA:RNA hybrids were eluted in 0.5% SDS. Eluted DNA:RNA hybrids were purified by phenol–chloroform extraction and quantified using a Qubit fluorometer (Thermo Fisher Scientific, #Q32854). Ten nanograms of DNA:RNA hybrids were used for downstream library preparation.

### Library preparation

For DRIP-Seq, 10 ng of immunoprecipitated DNA:RNA hybrids were sonicated into small fragments (∼100-500 bp) using Covaris Focused-Ultrasonicator (Me220) in a 25 μl reaction. After fragmentation, DNA was blunted, end-repaired, 5′ phosphorylated, and 3′ A-tailed using 1.5 μl of NEBNext Ultra II End Prep Enzyme Mix from NEBNext Ultra II DNA Library Prep Kit for Illumina (NEB, #E7645L) following the manufacturer's instructions. The diluted adaptor (1.25 μl) was ligated to the DNA based on optimized working concentration (1:10 dilution for 5-50 ng DNA; 1:15 dilution for <5 ng DNA). USER enzyme (3 μl) was used for U excision to yield the adaptor-ligated double-stranded DNA, followed by cleanup and size selection using AMPure XP beads (1:1 ratio, v/v) (Beckman Coulter, #A63881). Following the manufacturer's instructions, adaptor-ligated DNA was then PCR amplified using barcoded PCR primers (NEB, #E7335L, #E7500L). Following AMPure XP bead purification and Qubit quantification, library size and quality were assessed using the 2100 Bioanalyzer system and sent to Admera Health for sequencing. Twenty to 30 samples were pooled in one lane, and libraries were sequenced on a NovaSeq platform with a read length configuration of 150 bp pair-ends, targeting 2-2.5B total reads per lane (1-1.25B in each direction).

### RNA-Seq

RNA was isolated as described in the “RNA extraction” section. One microgram of total RNA was sent to Admera Health for library construction. Following the manufacturer's instructions, the KAPA RNA Hyperprep Kit with RiboErase (Roche, #KK8561) was used for rRNA depletion and library construction. qPCR was used to measure library concentration, and quality was evaluated using the Tapestation High Sensitivity D1000 ScreenTapes (Agilent Technologies). Libraries were sequenced on a NovaSeq platform with a read length configuration of 150 pair-end, targeting 80 M reads per sample (40 M in each direction).

### Bioinformatic analysis

DRIP-Seq reads were aligned to the human genome sequence (hg38) by Bowtie 2 (version 2.5.2) with default parameters (81). Aligned reads were sorted by genomic coordinate using samtools (version 1.19.2) (82). The genome was divided into 500 bp bins, and R-loop read counts were calculated for each bin. Bins with read counts >20 in all replicates of either C-NPCs or AT-NPCs were kept for differential analysis by DESeq2, and significantly differential bins were defined by FDR <0.05 (34) (Figs. 2, *A* and *B*; 4, *A*–*D*). Peak calling was performed using MACS2 (version 2.2.9.1) (83), with their corresponding input BAM files. Peaks from the three technical replicates of each condition (C-NPCs and AT-NPCs, UT and IR) were merged to generate condition-specific peak regions. These peak regions were then overlapped with the significantly differential bins to identify those with *bona fide* R-loop signals using bedtools intersect (version 2.31.1) with default parameters and the -wa option to report the overlapping bins (84). For each comparison (*e.g.*, UT AT-NPCs *versus* UT C-NPCs), bins upregulated in AT-NPCs were intersected with peak regions derived from UT AT-NPC replicates, whereas bins downregulated in AT-NPCs (*i.e.*, higher in C-NPCs) were overlapped with peak regions from UT C-NPC replicates. Upon IR, differential R-loop bins were identified by comparing IR *versus* UT conditions within each cell type (C-NPCs and AT-NPCs) as described above. Upregulated and downregulated bins from each comparison were then overlapped using bedtools intersect to identify shared IR-responsive bins as well as C-NPC- and AT-NPC-specific responses. All R-loop regions were annotated to genes using annotePeaks.pl from HOMER (version 4.11) (85).

RNA-Seq reads were aligned to the human genome (hg38) by TopHat (version 2.1.0) with default parameters (86). Aligned reads were sorted by genomic coordinate using samtools (version 1.19.2) (82). Differential gene expression analysis was conducted by Cuffdiff (version 2.2.1) (35). Significantly DEGs were defined by *p* < 0.05 (Figs. 2*D*; 5, *A* and *B*). To identify genes showing both transcriptional and R-loop changes, DEGs were overlapped with genes associated with differential R-loop regions.

GO analysis was performed by PANTHER (87). The background list used for each analysis was a list of 20,592 human genes available in the database. Protein–protein interaction networks were generated using the online tool STRING (36).

### Quantification and statistical analysis

Western blot and dot blot images were quantified using the Image Lab program (https://www.bio-rad.com/en-us/product/image-lab-software?ID=KRE6P5E8Z). Immunofluorescence images were quantified using the ImageJ program (https://imagej.net/ij/). Unpaired two-tailed Student's *t* test was performed in GraphPad Prism 9.5.1 to test statistical significances and obtain *p* values for RT–qPCR (Fig. 1*D*), Western blot quantification (Fig. 1, *E* and *F*; Fig. 3, *A* and *B*), dot blot quantification (Fig. 1*G*), and γH2A.X IF signal quantification (Fig. S1*C*). N and *p* values were indicated in the figure legends. N represents the number of replicates or genes. ∗*p* < 0.05; ∗∗*p* < 0.01; ∗∗∗*p* < 0.001; ∗∗∗∗*p* < 0.0001. All data points represent the mean ± SEM.

## Data availability

High-throughput sequencing data generated in this study, including DRIP-Seq and RNA-Seq, were deposited at Gene Expression Omnibus under accession numbers GSE304105 and GSE304101, respectively. Token for reviewer access to DRIP-Seq data is ejazmskojvetdwf. Token access to RNA-Seq data is yjmjcwcohlknroh. Original imaging and gel data have been deposited at Mendeley and are publicly available as of the date of publication. The DOI is 10.17632/stphcf47y2.2. Any additional information required to reanalyze the data reported in this article is available from the lead contact upon request.

## Supporting information

This article contains supporting information.

## Conflict of interest

The authors declare that they have no conflicts of interest with the contents of this article.
