# Supplementary material for: Loss of ATM causes R-loop–associated transcriptional dysregulation and attenuates the related response to DNA damage
Source: J Biol Chem. 2026 Jan 14;302(3):111161. doi: 10.1016/j.jbc.2026.111161 (PMC12906170; doi:10.1016/j.jbc.2026.111161)
Supplement: Figure S1-S6 [file mmc1.pdf]

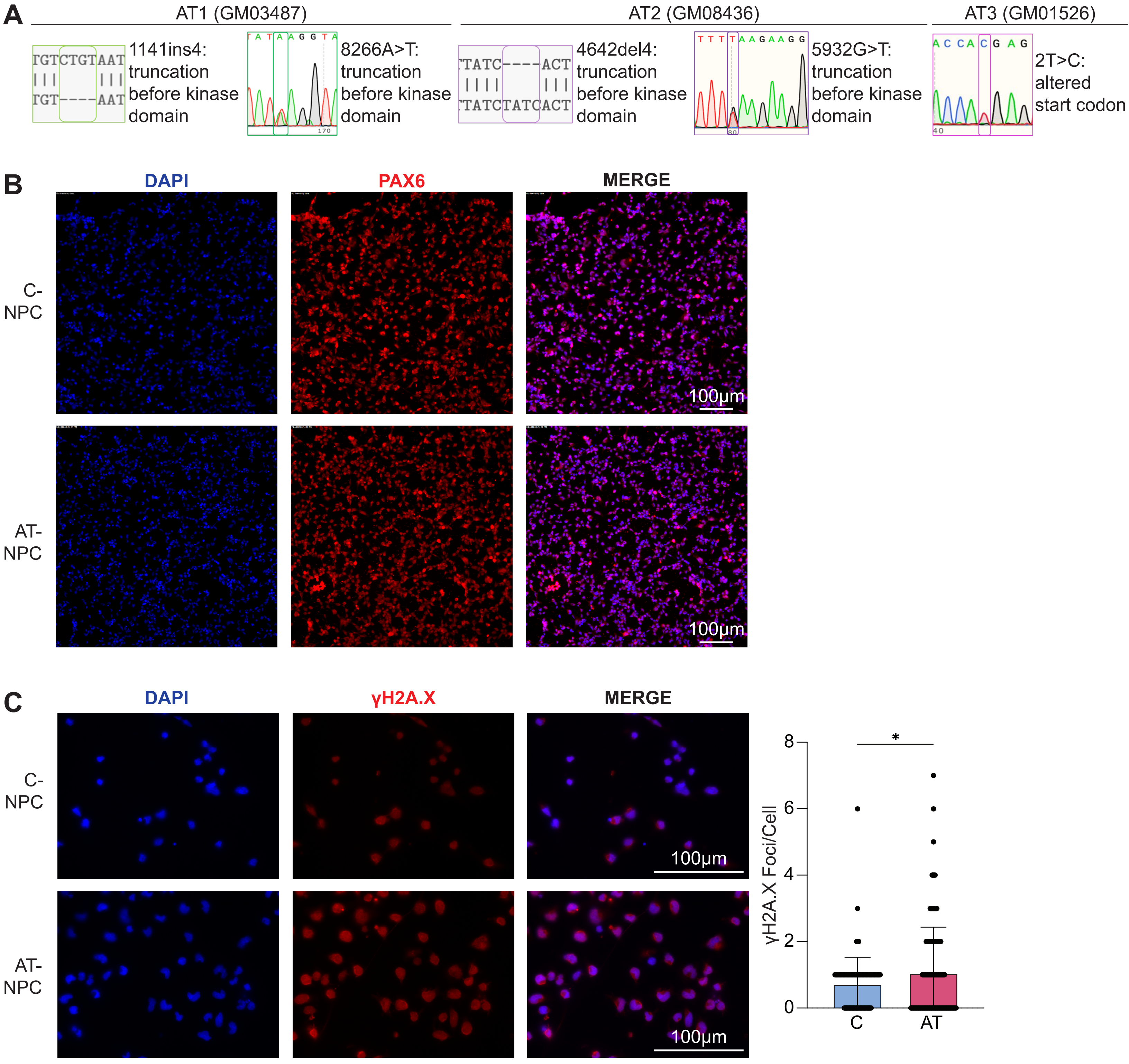
A
AT1 (GM03487)
1141ins4: truncation before kinase domain
8266A>T: truncation before kinase domain
AT2 (GM08436)
4642del4: truncation before kinase domain
5932G>T: truncation before kinase domain
AT3 (GM01526)
2T>C: altered start codon
B
DAPI
PAX6
MERGE
C-NPC
AT-NPC
100µm
C
DAPI
γH2A.X
MERGE
C-NPC
AT-NPC
100µm
γH2A.X Foci/Cell
*
C
AT

Figure S1

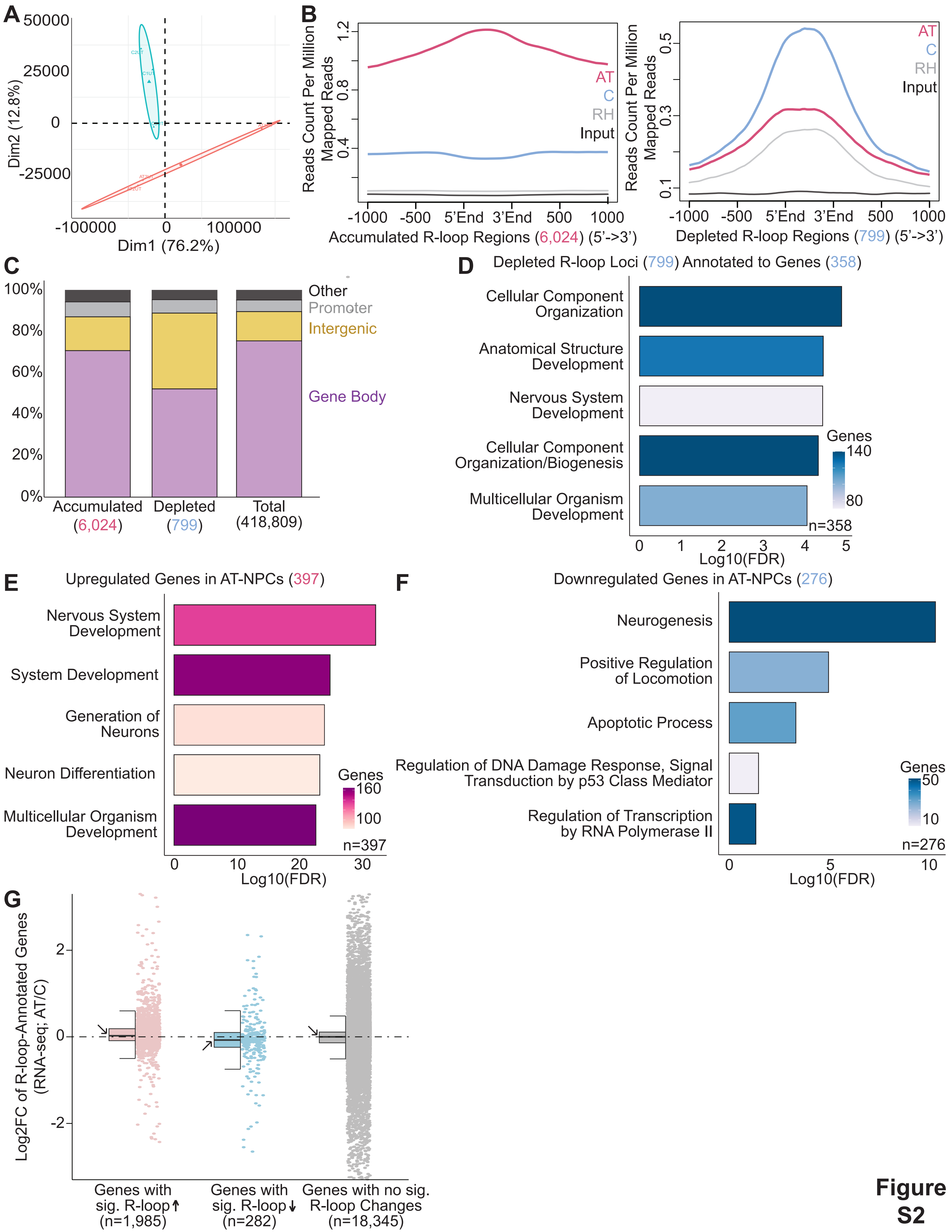
A
Dim1 (76.2%)
Dim2 (12.8%)
B
Reads Count Per Million Mapped Reads
AT
C
RH
Input
-1000 -500 5'End 3'End 500 1000
Accumulated R-loop Regions (6,024) (5'->3')
Depleted R-loop Regions (799) (5'->3')
C
Other
Promoter
Intergenic
Gene Body
Accumulated (6,024)
Depleted (799)
Total (418,809)
D
Depleted R-loop Loci (799) Annotated to Genes (358)
Cellular Component Organization
Anatomical Structure Development
Nervous System Development
Cellular Component Organization/Biogenesis
Multicellular Organism Development
Genes
n=358
Log10(FDR)
E
Upregulated Genes in AT-NPCs (397)
Nervous System Development
System Development
Generation of Neurons
Neuron Differentiation
Multicellular Organism Development
Genes
n=397
Log10(FDR)
F
Downregulated Genes in AT-NPCs (276)
Neurogenesis
Positive Regulation of Locomotion
Apoptotic Process
Regulation of DNA Damage Response, Signal Transduction by p53 Class Mediator
Regulation of Transcription by RNA Polymerase II
Genes
n=276
Log10(FDR)
G
Log2FC of R-loop-Annotated Genes (RNA-seq; AT/C)
Genes with sig. R-loop↑ (n=1,985)
Genes with sig. R-loop↓ (n=282)
Genes with no sig. R-loop Changes (n=18,345)

Figure S2

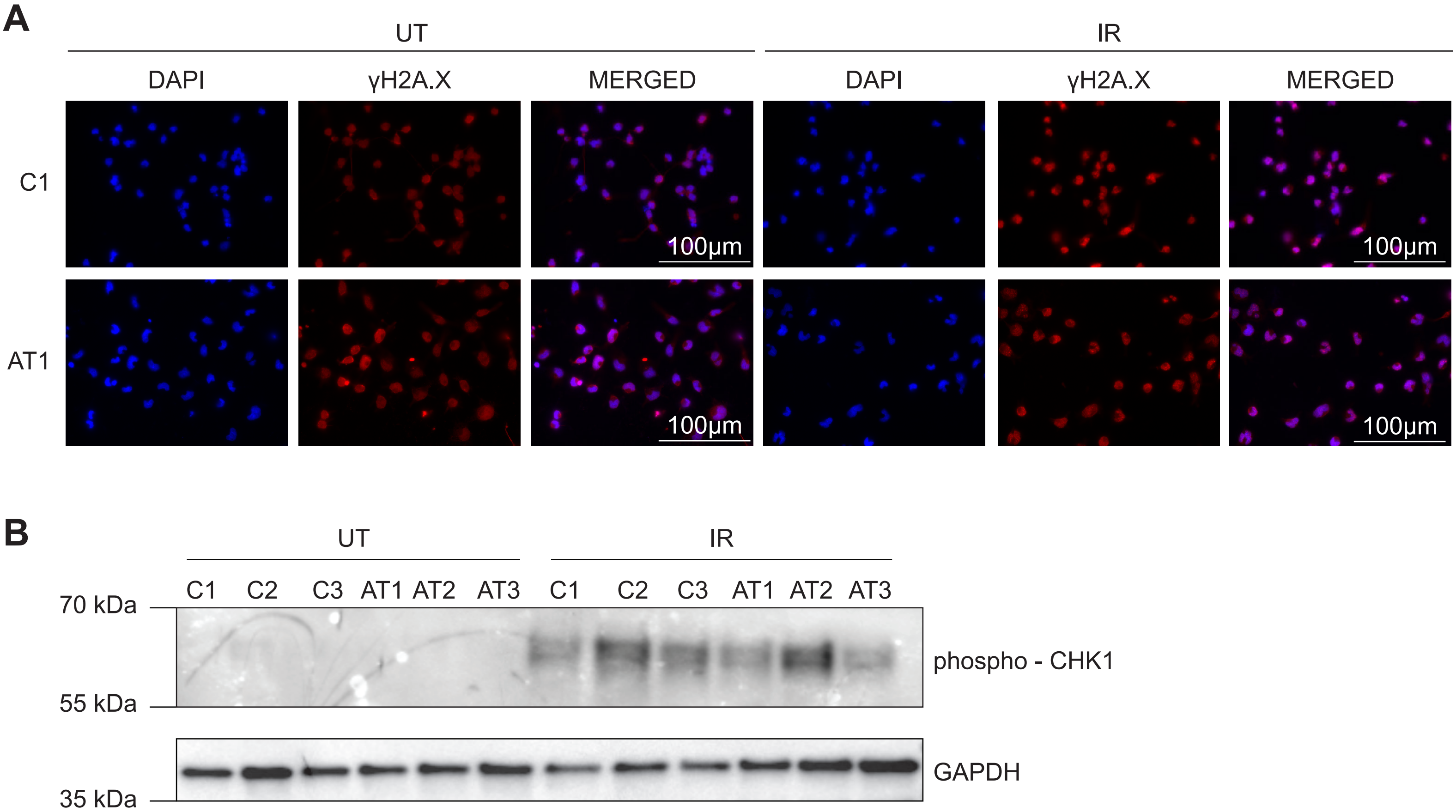
A
UT
IR
DAPI
γH2A.X
MERGED
DAPI
γH2A.X
MERGED
C1
AT1
100µm
100µm
100µm
100µm
B
UT
IR
C1 C2 C3 AT1 AT2 AT3 C1 C2 C3 AT1 AT2 AT3
70 kDa
55 kDa
35 kDa
phospho - CHK1
GAPDH

Figure S3

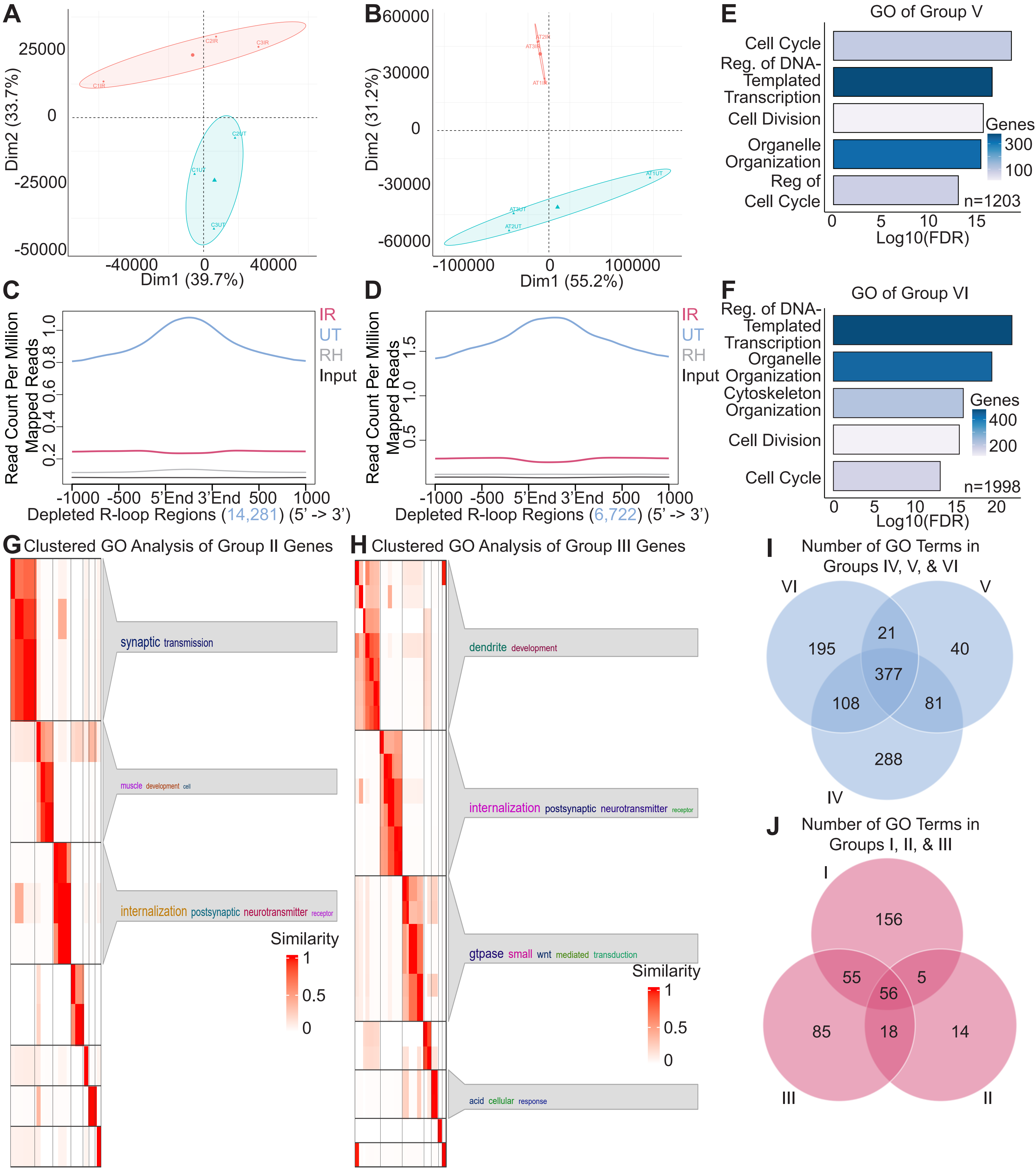
A
Dim2 (33.7%)
25000
0
-25000
-50000
-40000
0
40000
Dim1 (39.7%)
B
Dim2 (31.2%)
60000
30000
0
-30000
-60000
-100000
0
100000
Dim1 (55.2%)
E
GO of Group V
Cell Cycle
Reg. of DNA-Templated Transcription
Cell Division
Organelle Organization
Reg of Cell Cycle
Genes
300
100
n=1203
0
5
10
15
Log10(FDR)
C
Read Count Per Million Mapped Reads
IR
UT
RH
Input
-1000 -500 5'End 3'End 500 1000
Depleted R-loop Regions (14,281) (5' -> 3')
D
Read Count Per Million Mapped Reads
IR
UT
RH
Input
-1000 -500 5'End 3'End 500 1000
Depleted R-loop Regions (6,722) (5' -> 3')
F
GO of Group VI
Reg. of DNA-Templated Transcription
Organelle Organization
Cytoskeleton Organization
Cell Division
Cell Cycle
Genes
400
200
n=1998
0
5
10
15
20
Log10(FDR)
G
Clustered GO Analysis of Group II Genes
synaptic transmission
muscle development cell
internalization postsynaptic neurotransmitter receptor
Similarity
1
0.5
0
H
Clustered GO Analysis of Group III Genes
dendrite development
internalization postsynaptic neurotransmitter receptor
gtpase small wnt mediated transduction
acid cellular response
Similarity
1
0.5
0
I
Number of GO Terms in Groups IV, V, & VI
VI
V
IV
195
21
40
377
108
81
288
J
Number of GO Terms in Groups I, II, & III
I
III
II
156
55
5
56
85
18
14

Figure S4

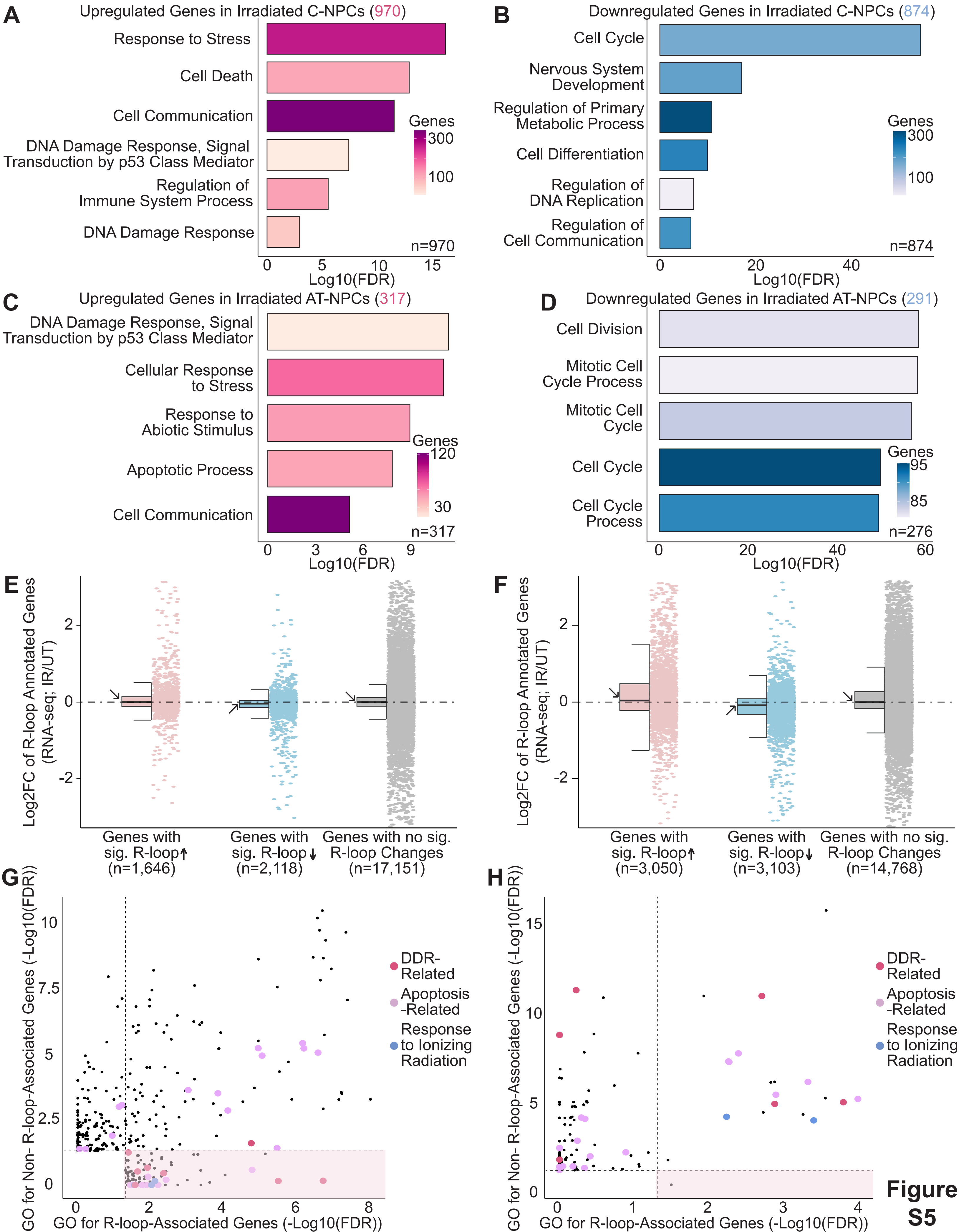
A
Upregulated Genes in Irradiated C-NPCs (970)
Response to Stress
Cell Death
Cell Communication
DNA Damage Response, Signal Transduction by p53 Class Mediator
Regulation of Immune System Process
DNA Damage Response
Genes
300
100
n=970
0
5
10
15
Log10(FDR)
B
Downregulated Genes in Irradiated C-NPCs (874)
Cell Cycle
Nervous System Development
Regulation of Primary Metabolic Process
Cell Differentiation
Regulation of DNA Replication
Regulation of Cell Communication
Genes
300
100
n=874
0
20
40
Log10(FDR)
C
Upregulated Genes in Irradiated AT-NPCs (317)
DNA Damage Response, Signal Transduction by p53 Class Mediator
Cellular Response to Stress
Response to Abiotic Stimulus
Apoptotic Process
Cell Communication
Genes
120
30
n=317
0
3
6
9
Log10(FDR)
D
Downregulated Genes in Irradiated AT-NPCs (291)
Cell Division
Mitotic Cell Cycle Process
Mitotic Cell Cycle
Cell Cycle
Cell Cycle Process
Genes
95
85
n=276
0
20
40
60
Log10(FDR)
E
Log2FC of R-loop Annotated Genes (RNA-seq; IR/UT)
2
0
-2
Genes with sig. R-loop↑ (n=1,646)
Genes with sig. R-loop↓ (n=2,118)
Genes with no sig. R-loop Changes (n=17,151)
F
Log2FC of R-loop Annotated Genes (RNA-seq; IR/UT)
2
0
-2
Genes with sig. R-loop↑ (n=3,050)
Genes with sig. R-loop↓ (n=3,103)
Genes with no sig. R-loop Changes (n=14,768)
G
GO for Non- R-loop-Associated Genes (-Log10(FDR))
10
7.5
5
2.5
0
DDR-Related
Apoptosis-Related
Response to Ionizing Radiation
0
2
4
6
8
GO for R-loop-Associated Genes (-Log10(FDR))
H
GO for Non- R-loop-Associated Genes (-Log10(FDR))
15
10
5
0
DDR-Related
Apoptosis-Related
Response to Ionizing Radiation
0
1
2
3
4
GO for R-loop-Associated Genes (-Log10(FDR))

Figure S5

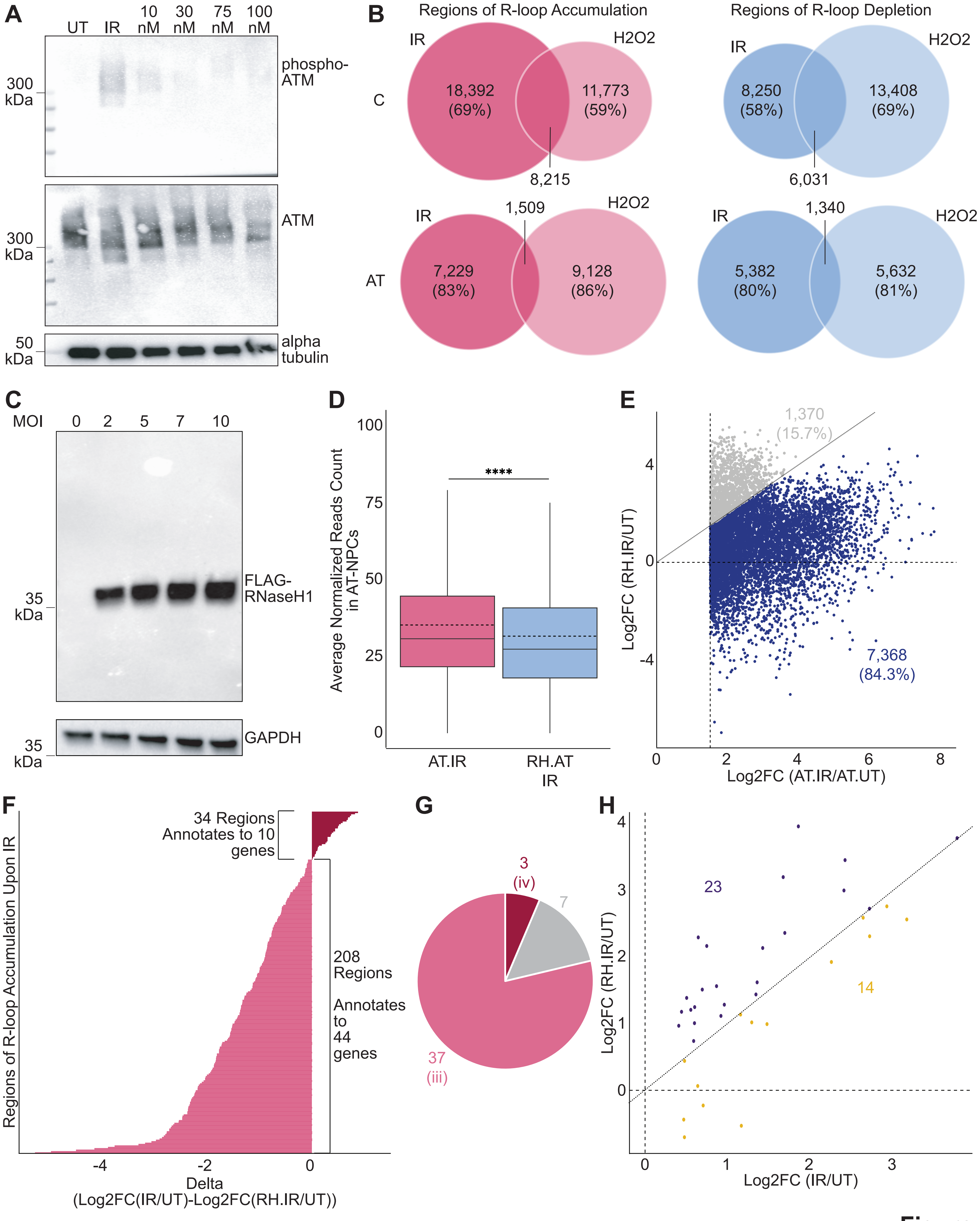

A
UT
IR
10 nM
30 nM
75 nM
100 nM
300 kDa
phospho-ATM
ATM
50 kDa
alpha tubulin
B
Regions of R-loop Accumulation
Regions of R-loop Depletion
C
AT
IR
H2O2
18,392 (69%)
11,773 (59%)
8,215
8,250 (58%)
13,408 (69%)
6,031
1,509
7,229 (83%)
9,128 (86%)
1,340
5,382 (80%)
5,632 (81%)
C
MOI
0
2
5
7
10
35 kDa
FLAG-RNaseH1
GAPDH
D
Average Normalized Reads Count in AT-NPCs
****
AT.IR
RH.AT IR
E
1,370 (15.7%)
7,368 (84.3%)
Log2FC (RH.IR/UT)
Log2FC (AT.IR/AT.UT)
F
34 Regions Annotates to 10 genes
208 Regions Annotates to 44 genes
Regions of R-loop Accumulation Upon IR
Delta (Log2FC(IR/UT)-Log2FC(RH.IR/UT))
G
3 (iv)
7
37 (iii)
H
23
14
Log2FC (RH.IR/UT)
Log2FC (IR/UT)

Figure S6

Supplemental Figure 1. Loss of ATM protein in AT patient-derived NPCs (AT-NPCs) results in a global increase in DNA damage and R-loops. *A,* Validation of AT patient mutations through Sanger sequencing. GM03487 (AT1; left) and GM08436 (AT2; middle) are compound heterozygotes containing two separate mutations. GM01526 (AT3; right) contains one mutation. *B,* Representative immunofluorescence images of C- (top) and AT-NPC (bottom) differentiation. Cells were stained for DAPI (blue) and NPC marker PAX6 (red). Scale bar: 100 µm. *C,* Representative immunofluorescence images for DNA damage marker γH2A.X (red) staining in C- (top) and AT-NPCs (bottom). Scale bar: 100 µm. Quantification (right) of the number of γH2A.X foci was counted in representative images. All data plotted as mean ± SEM. *$p < 0.05$.

Supplemental Figure 2. AT-NPCs demonstrate a higher accumulation of R-loops which correlates with upregulation of genes involved in neuronal function and DNA damage response. *A,* PCA analysis of DRIP-seq data for C- and AT-NPCs. C-NPCs (blue) and AT-NPCs (red) cluster separately (n = 3). *B,* Metagene plots using ngs.plot of R-loop reads over the 6,024 accumulated (left) and 799 depleted (right) regions. *C,* Proportion of accumulated (left), depleted (middle), and total (right) R-loop regions annotated to gene bodies (purple), intergenic regions (gold), promoters (grey), and other regions (black). *D,* GO analysis of genes (n = 358) associated with loci with significant R-loop depletion (n = 799) in AT-NPCs. Bar color indicates number of genes associated with each GO term. *E,* GO analysis of upregulated genes (n = 397) in AT-NPCs. Bar color indicates number of genes associated with each GO term. *F,* GO analysis of downregulated genes (n = 276) in AT-NPCs. Bar color indicates number of genes associated with each GO term. *G,* Half-jitter plot analysis of differential gene expression associated with changes in R-loop regions. Dots indicate the expression changes of genes annotated by differential R-loops and boxplots show median and quartiles in each category. Genes with significant R-loop accumulation are shown in pink (n = 1,985), genes with significant R-loop depletion in blue (n = 282) and genes with no significant R-loop changes in grey (n = 18,345).

Supplemental Figure 3. Irradiation induces accumulation of DNA damage and activation of ATM. *A,* Representative immunofluorescence staining for DAPI (blue) and DNA damage marker γH2A.X (red) in C- (top) and AT-NPCs (bottom) that are untreated (UT; left) and irradiated (IR; right). Scale bar: 100 µm. *B*, Western blot analysis of S345 phosphorylated CHK1 protein levels (top) in UT and IR C- and AT-NPCs. GAPDH protein levels (bottom) were used as loading controls.

Supplemental Figure 4. AT-NPCs demonstrate an impaired R-loop response to irradiation. *A,* PCA analysis of DRIP-seq data for untreated (UT; blue) and irradiated (IR; red) C-NPCs (n = 3). *B,* PCA analysis of DRIP-seq data for UT and IR AT-NPCs (n = 3). *C,* Metagene plot using ngs.plot of R-loop reads over the 14,281 R-loop depleted regions in C-NPCs upon irradiation. *D,* Metagene plot using ngs.plot of R-loop reads over the 6,722 R-loop depleted regions in AT-NPCs upon irradiation. *E,* GO analysis of Fig 4G Group V genes (n = 1,203). Bar color indicates number of genes associated with each GO term. *F,* GO analysis of Fig 4G Group VI genes (n = 1998). Bar color indicates number of genes associated with each GO term. *G,* Clustered gene ontology (GO) analysis of Group II genes (n = 794) in Fig 4G. Word size indicates GO term enrichment. *H,* Clustered GO analysis of Fig 4G Group III genes (n = 1,486). Word size indicates GO term enrichment. *I,* Venn diagram of the number of GO terms unique and shared between Fig 4G Groups IV, V, and VI. *J,* Venn diagram of the number of GO terms unique and shared between Fig 4G Groups I, II, and III.

Supplemental Figure 5. AT-NPCs lack R-loop-associated changes in transcription in DDR related genes. *A,* Gene ontology (GO) analysis of genes upregulated (n = 970) upon irradiation in C-NPCs. Bar color indicates number of genes associated with each GO term. *B,* GO analysis of genes downregulated (n = 874) upon irradiation in C-NPCs. Bar color indicates number of genes associated with each GO term. *C,* GO analysis of genes upregulated (n = 317) upon irradiation in AT-NPCs. Bar color indicates number of genes associated with each GO term. *D,* GO analysis of genes downregulated (n = 291) upon irradiation in AT-NPCs. Bar color indicates number of genes associated with each GO term. *E,* Half-jitter plot analysis of differential gene expression associated with changes in R-loop regions upon irradiation in AT-NPCs. Dots indicate the expression changes of genes annotated by differential R-loops and boxplots show median and quartiles in each category. Genes with significant R-loop accumulation are shown in pink (n = 3,050), genes with significant R-loop depletion in blue (n = 3,103) and genes with no significant R-loop changes in grey (n = 14,768). *F,* Half-jitter plot analysis of differential gene expression associated with changes in R-loop regions upon irradiation in C-NPCs. Dots indicate the expression changes of genes annotated by differential R-loops and boxplots show median and quartiles in each category. Genes with significant R-loop accumulation are shown in pink (n = 1,646), genes with significant R-loop depletion in blue (n = 2,118) and genes with no significant R-loop changes in grey (n = 16,931). *G,* Comparison of significant GO terms (FDR < 0.05) for upregulated genes with significant R-loop accumulation in C-NPCs (n of GO terms = 214, x-axis) and upregulated genes with no significant R-loop association (n of GO

terms = 269; y-axis). GO terms relating to the DNA damage response, apoptosis, and response to ionizing radiation highlighted are labeled in red, purple, and blue, respectively. GO terms significantly enriched only for R-loop associated genes are highlighted by the pink box. *H,* Comparison of significant GO terms (FDR < 0.05) for genes with significant upregulation and R-loop accumulation in AT-NPCs (n of GO terms = 20, x-axis) and upregulated genes with no significant R-loop association (n of GO terms = 148; y-axis). GO terms relating to the DNA damage response, apoptosis, and response to ionizing radiation are highlighted in red, purple, and blue, respectively. GO terms significantly enriched only for R-loop associated genes are highlighted by the pink box.

Supplemental Figure 6. R-loops play broad and causal roles in responding to DNA damage induced from diverse sources, by regulating the expression of a subset of key DDR genes. *A*, Western blot of S1981 phosphorylated ATM (top) and non-phosphorylated ATM (middle) in C-NPCs treated with differing concentrations of the ATM inhibitor AZD1390 (10nM, 30nM, 75nM, and 100nM). *B*, Venn diagrams of R-loop responses to irradiation (IR, left circle) and H2O2 treatment (right circle) in C- (top row) and AT-NPCs (bottom row). Regions of R-loop accumulation on the left and regions of R-loop depletion on the right. *C*, Western blot of FLAG-tagged RNaseH1 overexpression in NPCs infected with different MOIs. *D*, Boxplot of average normalized reads counts (DESeq2, n = 3; Student's t-test) in irradiated AT-NPCs (IR; pink) and irradiated NPCs overexpressing RNaseH1 (RH.IR; blue). *E*, Scatterplot of Log_2FC of DRIP-seq results (DESeq2, n = 3) in regions of R-loop accumulation in IR (Log_2FC (IR/UT); x-axis) and RH.IR cells (Log_2FC (RH.IR/UT); y-axis). Blue dots indicate regions of dampened R-loop accumulation in response to irradiation in RH.IR cells (7,368 regions, 84.3%; Log_2FC (RH.IR/UT) < Log_2FC (IR/UT)). Grey dots indicate regions of increased R-loop accumulation in response to irradiation in RH.IR cells (1,370 regions, 15.7%; Log_2FC (RH.IR/UT) > Log_2FC (IR/UT)). *F*, Bar plot of the difference between Log_2FC (RH.IR/UT) and Log_2FC (IR/UT) in regions of significant R-loop accumulation upon irradiation in AT-NPCs which annotate to upregulated genes (n = 242 regions; delta = Log_2FC (IR/UT) – Log_2FC (RH.IR/UT), x-axis). Pink bars indicate regions of dampened R-loop accumulation in RH.IR cells (208 regions annotating to 44 genes; delta < 0). Red bars indicate regions of increased R-loop accumulation in RH.IR cells (34 regions annotating to 10 genes; delta > 0). *G*, Pie chart of genes identified in (*F*). Pink indicates genes which contain regions of dampened R-loop accumulation upon irradiation in RH.IR cells (n = 37; Group iii). Red indicates genes which contain regions of increased R-loop accumulation upon irradiation in RH.IR cells (n = 3, Group iv). Grey indicates genes which contain both regions of dampened accumulation and regions of increased

accumulation (n = 7). *H*, Scatterplot of RNA-seq results (CuffDiff, n = 3) in genes with dampened R-loop accumulation upon RNaseH1 overexpression in IR (Log_2FC (IR/UT); x-axis) and RH.IR (Log_2FC (RH.IR/UT); y-axis). Gold dots indicate genes which contain regions of dampened R-loop accumulation and dampened gene upregulation upon irradiation in RH.IR cells (n = 14; Log_2FC (RH.IR/UT) < Log_2FC (IR/UT)). Purple dots indicate genes which contain regions of dampened R-loop accumulation yet maintain upregulation of gene expression to levels observed in IR cells (n = 23; Log_2FC (RH.IR/UT) > Log_2FC (IR/UT)). All data plotted as mean ± SEM. ****p < 0.0001.
